# Artificial intelligence defines protein-based classification of thyroid nodules

**DOI:** 10.1038/s41421-022-00442-x

**Published:** 2022-09-06

**Authors:** Yaoting Sun, Sathiyamoorthy Selvarajan, Zelin Zang, Wei Liu, Yi Zhu, Hao Zhang, Wanyuan Chen, Hao Chen, Lu Li, Xue Cai, Huanhuan Gao, Zhicheng Wu, Yongfu Zhao, Lirong Chen, Xiaodong Teng, Sangeeta Mantoo, Tony Kiat-Hon Lim, Bhuvaneswari Hariraman, Serene Yeow, Syed Muhammad Fahmy Alkaff, Sze Sing Lee, Guan Ruan, Qiushi Zhang, Tiansheng Zhu, Yifan Hu, Zhen Dong, Weigang Ge, Qi Xiao, Weibin Wang, Guangzhi Wang, Junhong Xiao, Yi He, Zhihong Wang, Wei Sun, Yuan Qin, Jiang Zhu, Xu Zheng, Linyan Wang, Xi Zheng, Kailun Xu, Yingkuan Shao, Shu Zheng, Kexin Liu, Ruedi Aebersold, Haixia Guan, Xiaohong Wu, Dingcun Luo, Wen Tian, Stan Ziqing Li, Oi Lian Kon, Narayanan Gopalakrishna Iyer, Tiannan Guo

**Affiliations:** 1 Westlake Laboratory of Life Sciences and Biomedicine, Key Laboratory of Structural Biology of Zhejiang Province, School of Life Sciences, Westlake University, Hangzhou, Zhejiang China; 2 Institute of Basic Medical Sciences, Westlake Institute for Advanced Study, Hangzhou, Zhejiang China; 3 Research Center for Industries of the Future, Westlake University, No.18 Shilongshan Road, Hangzhou, Zhejiang China; 4 Department of Anatomical Pathology, Division of Pathology, Singapore General Hospital, Singapore, Singapore; 5 School of Engineering, Westlake University, No.18 Shilongshan Road, Hangzhou, Zhejiang China; 6 Westlake Omics (Hangzhou) Biotechnology Co., Ltd., No.1 Yunmeng Road, Hangzhou, Zhejiang China; 7 Department of Thyroid Surgery, the First Hospital of China Medical University, Shenyang, Liaoning China; 8 Cancer Center, Department of Pathology, Zhejiang Provincial People’s Hospital, Affiliated People’s Hospital, Hangzhou Medical College, Hangzhou, Zhejiang China; 9 Department of General Surgery, The Second Hospital of Dalian Medical University, Dalian, Liaoning China; 10 Department of Pathology, The Second Affiliated Hospital of College of Medicine, Zhejiang University, Hangzhou, Zhejiang China; 11 Department of Pathology, the First Affiliated Hospital, College of Medicine, Zhejiang University, Hangzhou, Zhejiang China; 12 Department of Head and Neck Surgery, National Cancer Center Singapore, Singapore, Singapore; 13 Division of Medical Sciences, National Cancer Center Singapore, Singapore, Singapore; 14 Department of Surgical Oncology, the First Affiliated Hospital, College of Medicine, Zhejiang University, Hangzhou, Zhejiang China; 15 Department of Urology, The Second Hospital of Dalian Medical University, Dalian, Liaoning China; 16 Department of Ultrasound, Sir Run Run Shaw Hospital, Zhejiang University School of Medicine, Hangzhou, Zhejiang China; 17 Liaoning Laboratory of Cancer Genetics and Epigenetics and Department of Cell Biology, College of Basic Medical Sciences, Dalian Medical University, Dalian, Liaoning China; 18 Department of Ophthalmology, The Second Affiliated Hospital, Zhejiang University School of Medicine, Hangzhou, Zhejiang China; 19 Cancer Institute (Key Laboratory of Cancer Prevention and Intervention, China National Ministry of Education, Key Laboratory of Molecular Biology in Medical Sciences, Zhejiang, China), The Second Affiliated Hospital, Zhejiang University School of Medicine, Hangzhou, Zhejiang China; 20 Department of Clinical Pharmacology, College of Pharmacy, Dalian Medical University, Dalian, Liaoning China; 21 Department of Biology, Institute of Molecular Systems Biology, ETH Zurich, Zurich, Switzerland; 22 Faculty of Science, University of Zurich, Zurich, Switzerland; 23 Department of Endocrinology, Guangdong Provincial People’s Hospital, Guangdong Academy of Medical Sciences, Guangzhou, Guangdong China; 24 Department of Endocrinology, Zhejiang Provincial People’s Hospital, Affiliated People’s Hospital, Hangzhou, Zhejiang China; 25 Department of Surgical Oncology, Affiliated Hangzhou First People’s Hospital, Zhejiang University School of Medicine, Hangzhou, Zhejiang China; 26 Department of General Surgery, PLA General Hospital, Beijing, China; 27 Westlake Laboratory of Life Sciences and Biomedicine, Westlake University, Hangzhou, Zhejiang China

**Keywords:** Proteomics, Cancer models

## Abstract

Determination of malignancy in thyroid nodules remains a major diagnostic challenge. Here we report the feasibility and clinical utility of developing an AI-defined protein-based biomarker panel for diagnostic classification of thyroid nodules: based initially on formalin-fixed paraffin-embedded (FFPE), and further refined for fine-needle aspiration (FNA) tissue specimens of minute amounts which pose technical challenges for other methods. We first developed a neural network model of 19 protein biomarkers based on the proteomes of 1724 FFPE thyroid tissue samples from a retrospective cohort. This classifier achieved over 91% accuracy in the discovery set for classifying malignant thyroid nodules. The classifier was externally validated by blinded analyses in a retrospective cohort of 288 nodules (89% accuracy; FFPE) and a prospective cohort of 294 FNA biopsies (85% accuracy) from twelve independent clinical centers. This study shows that integrating high-throughput proteomics and AI technology in multi-center retrospective and prospective clinical cohorts facilitates precise disease diagnosis which is otherwise difficult to achieve by other methods.

## Introduction

Advances in imaging technology and liberal screening practices have identified thyroid nodules in up to 50% of the general population, but only a small minority of these (7%–15%) eventually prove to be malignant by histology, and an even smaller fraction is clinically relevant^1,2^. Beyond clinical assessment and ultrasonography, fine-needle aspiration (FNA) followed by cytopathology is considered the most reliable pre-surgical technique for differentiating benign from malignant thyroid tumors^1,3^. Yet up to one-third of thyroid nodules are deemed indeterminate by FNA-cytopathology^4^, and surgery remains the only option for accurate diagnosis. The majority of thyroid surgeries are diagnostic procedures undertaken to exclude thyroid cancers, of which ≤ 25% accomplish any therapeutic purpose^5^. Patients whose thyroid glands are removed in part or entirely often require daily and lifelong thyroxine-replacement therapy and medical monitoring. Given that only ~10% of resected glands prove to be malignant, the current clinical approach results in substantial over-treatment with unwarranted surgical risks for patients who could otherwise be treated conservatively^6^.

Molecular tests adjunctive to FNA-cytopathology have focused on RNA expression or DNA mutational profiling of aspirates obtained prior to surgery, using small quantities of RNA or DNA that can be amplified^7–10^. The development of a nucleic acid-based classifier has been a remarkable decade-long practice across multiple centers using various technologies. However, nucleic acid-based testing has its inherent limitations, i.e., the need for fresh tissue samples with undegraded RNA. Furthermore, thyroid tumors are usually indolent and nonlethal, harboring few gene alterations. While nucleic acid-based approaches continue to be refined, for example with successive iterations of ThyroSeq panels, there is an evident need for alternative approaches to address this diagnostic dilemma.

Until recently, proteomics-based analyses were limited to large tissue quantities and fresh/snap-frozen samples. Proteotyping hundreds of biopsy-level tissue samples from clinical cohorts remains unachievable with conventional methods. We have developed a pressure cycling technology (PCT) protocol for proteomic analysis of tissue biopsy samples^11^ which can be performed on minimal amounts of fresh-frozen tissue samples^12,13^. The method was recently extended to generate high-quality proteome data from biopsy-level formalin-fixed, paraffin-embedded (FFPE) tissue samples^14^. Samples prepared by PCT can be analyzed by a data-independent acquisition mass spectrometry (DIA-MS) method^15,16^, enabling practical proteomic analysis of biopsy-level FFPE, fresh-frozen or even cytopathologic (from needle biopsies) tissue samples at high throughput. We have furthermore shown that, in comparison to RNA samples, protein samples are substantially less prone to spontaneous degradation in clinical samples^17^. In this study, we applied PCT-DIA to analyze tissue samples from > 1000 patients and show that the high-quality proteotype data in conjunction with machine learning approaches identified a robust panel of protein markers that could be used to stratify thyroid diseases.

## Results

### Study design and clinical characteristics

**Fig. 1:**
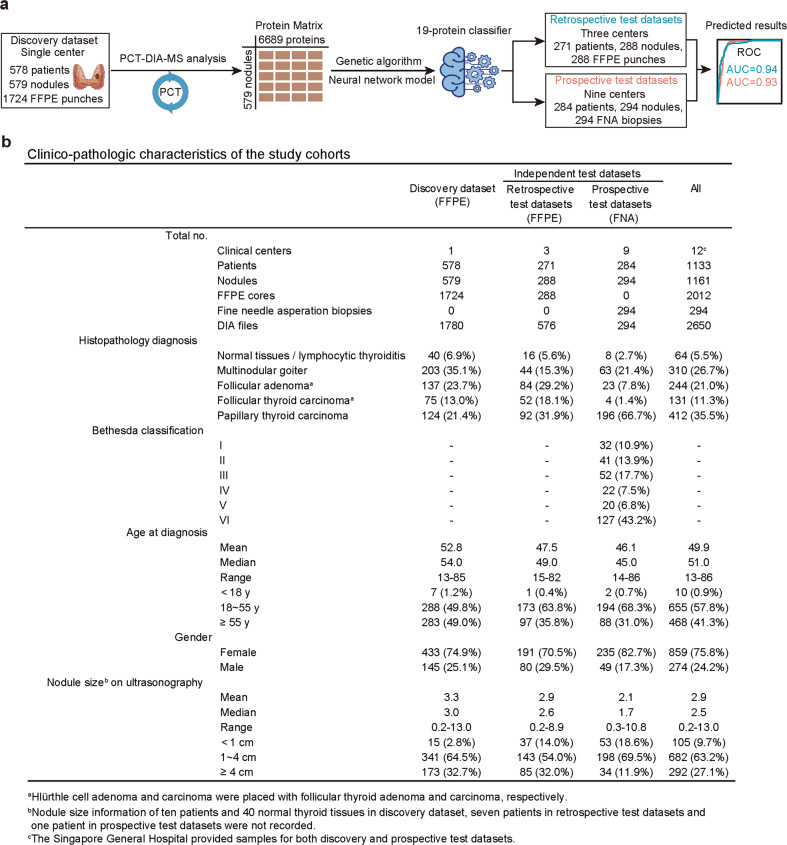
Schematic view of the study and clinic-pathologic characteristics. **a** The project design and workflow of the FFPE-PCT-DIA pipeline. **b** Clinic-pathologic characteristics of the study cohorts.

We applied PCT-DIA on a total of 1161 nodules from 1133 patients using either tissue cores (1 mm diameter; 0.5–1 mm depth) punched from regions of interest marked on retrospective FFPE tissue blocks or prospective cytology specimens from FNA aspirates. The samples comprise (i) a discovery set of FFPE samples from Singapore General Hospital (*n* = 579 nodules) where histopathological diagnoses were confirmed on central review by a board-certified pathologist; and independent test sets from twelve hospitals in China and Singapore consisting of (ii) retrospective test sets of FFPE samples (*n* = 288 nodules) with the same histopathological assessment and classification as the discovery sample set, and (iii) a prospective test set of FNA biopsies (*n* = 294 nodules) which were additionally scored by the Bethesda System for reporting thyroid cytopathology (Fig. 1a). Histological diagnoses of all tissue samples were based on uniform criteria^18^.

The discovery set comprised FFPE samples from 40 normal thyroid tissues (N), 203 multinodular goiters (MNG), 137 follicular thyroid adenomas (FA), 75 follicular thyroid carcinomas (FTC), and 124 papillary thyroid carcinomas (PTC) (Fig. 1b; Supplementary Table S1). For subsequent analyses, these samples were divided into benign (comprising N, MNG and FA) and malignant (comprising FTC and PTC) thyroid nodules. For each nodule in the discovery set, three cores were punched from the region of interest as replicates. We analyzed 1724 samples randomly distributed into 121 batches to minimize batch effects (Supplementary Fig. S1a) using 45-min DIA-MS. An additional 56 samples were randomly selected from the discovery dataset and used as technical replicates, i.e., injected into the mass spectrometer for DIA-MS analysis. Although greater proteomic depth could be obtained with a longer liquid chromatography (LC) gradient, we adopted a reasonably short analysis time to minimize batch effects without substantial compromise of proteome depth by taking advantage of the DIA-MS methodology, thus facilitating effective downstream machine learning to establish a robust classifier.

### Global proteomic profiling of thyroid nodules

To analyze the DIA data, we built a thyroid-specific spectral library from FFPE tissues as we described previously^19^. The library contained 925,330 transition groups, 157,548 peptide precursors, 121,960 peptides, 9941 protein groups, and 9826 proteins from proteotypic peptides. Using DIA-NN (v1.7.15) and our thyroid library, we analyzed 1780 DIA maps from 1724 FFPE cores and 56 aliquots of the same peptides injected as technical replicate samples for analysis by DIA-MS at specified points during data acquisition. We identified and quantified 63,036 peptides from 6749 protein groups, of which 6689 were proteotypic proteins in the discovery dataset (Supplementary Table S2). Details on quality control (QC) and reproducibility (Supplementary Fig. S2) are documented in the Materials and Methods.

**Fig. 2:**
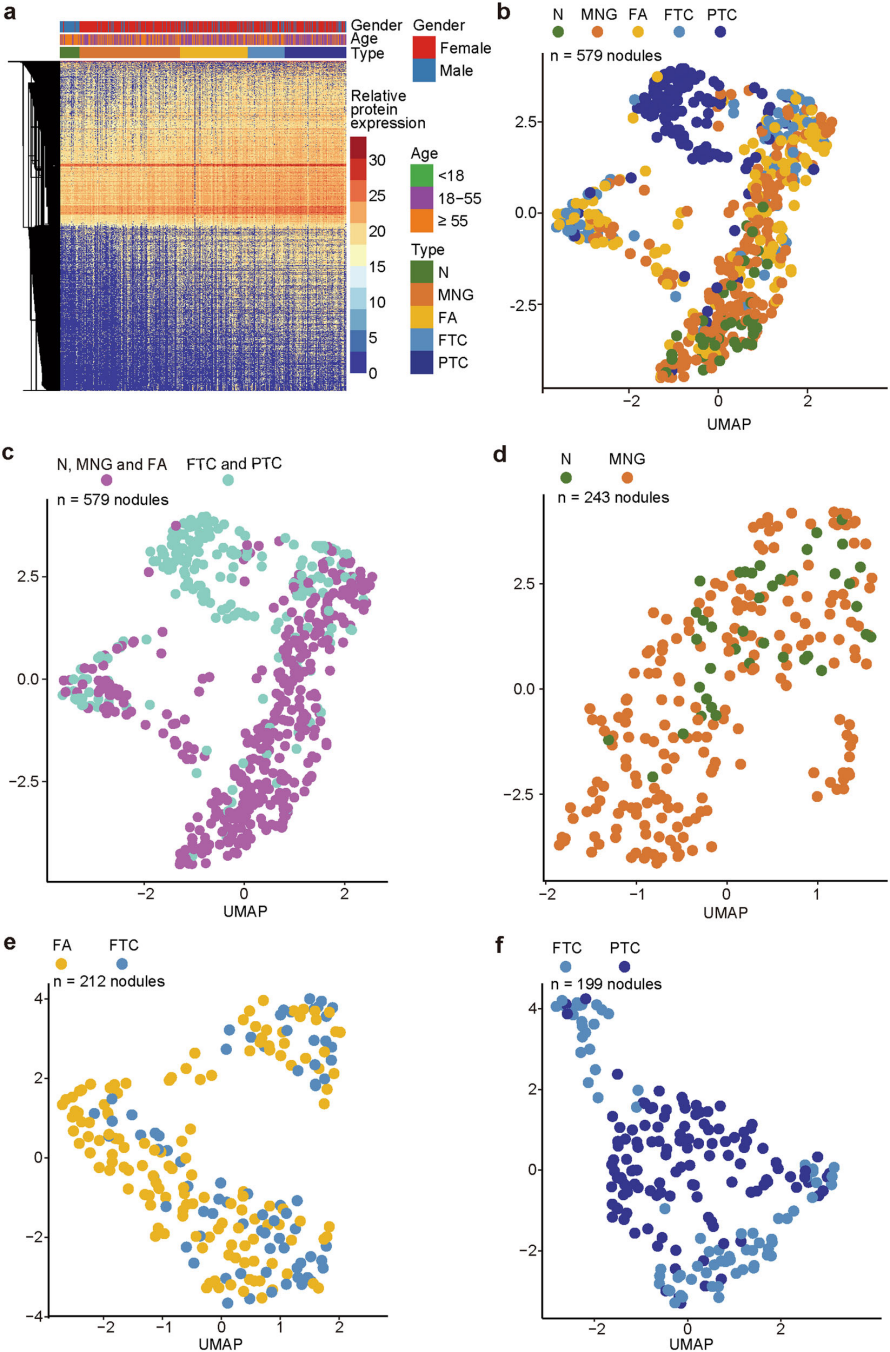
Global thyroid proteome profile. **a** Heatmap showing protein expression profiles of 579 thyroid tissue specimens from 578 patients. 5312 proteins (rows) are clustered without supervision. Samples (columns) are ordered based on the tissue types. The color indicates the log_2_-scaled intensity of each protein in each sample. **b**–**f** UMAP plots showing global snapshots comparing the indicated types of thyroid tissues using 5312 proteins for all subtypes (**b**); benign vs malignant (**c**); only benign (**d**); FA vs FTC (**e**); and only malignant (**f**) tissue types.

From these primary data, we computed the average intensities of 5312 proteotypic proteins which were quantified with < 90% missing values for each thyroid nodule, as visualized in a tissue-type arranged heatmap (Fig. 2a). Generally, a higher number of proteins were identified in malignant tissue samples compared to benign samples using the same amount of total peptide injected. Visualization of these data using uniform manifold approximation and projection (UMAP) plots showed that the PTC samples were well resolved from the rest. However, the N and MNG samples could not be separated from each other, neither could FA and FTC (Fig. 2b). We then grouped N, MNG, and FA as benign; FTC and PTC as malignant. These two groups are not completely separated in the UMAP analysis (Fig. 2c). We further narrowed our focus on the N and MNG samples and found that their proteotypes shared a high degree of similarity (Fig. 2d). Not surprisingly, FA could not be separated from benign or malignant subsets, particularly between FA and FTC (Fig. 2e), corroborating known biological similarities between these two pathologies which are believed to be part of a spectrum of follicular neoplasms. In contrast, there were sufficient features that distinguished FTC from PTC (Fig. 2f). Pairwise comparisons of each combination of two histological types are shown in Supplementary Fig. S3. The foregoing analyses showed that the proteotype maps thus measured reasonably reflected the histopathological phenotypes of these samples.

### Feature selection and classifier development

**Fig. 3:**
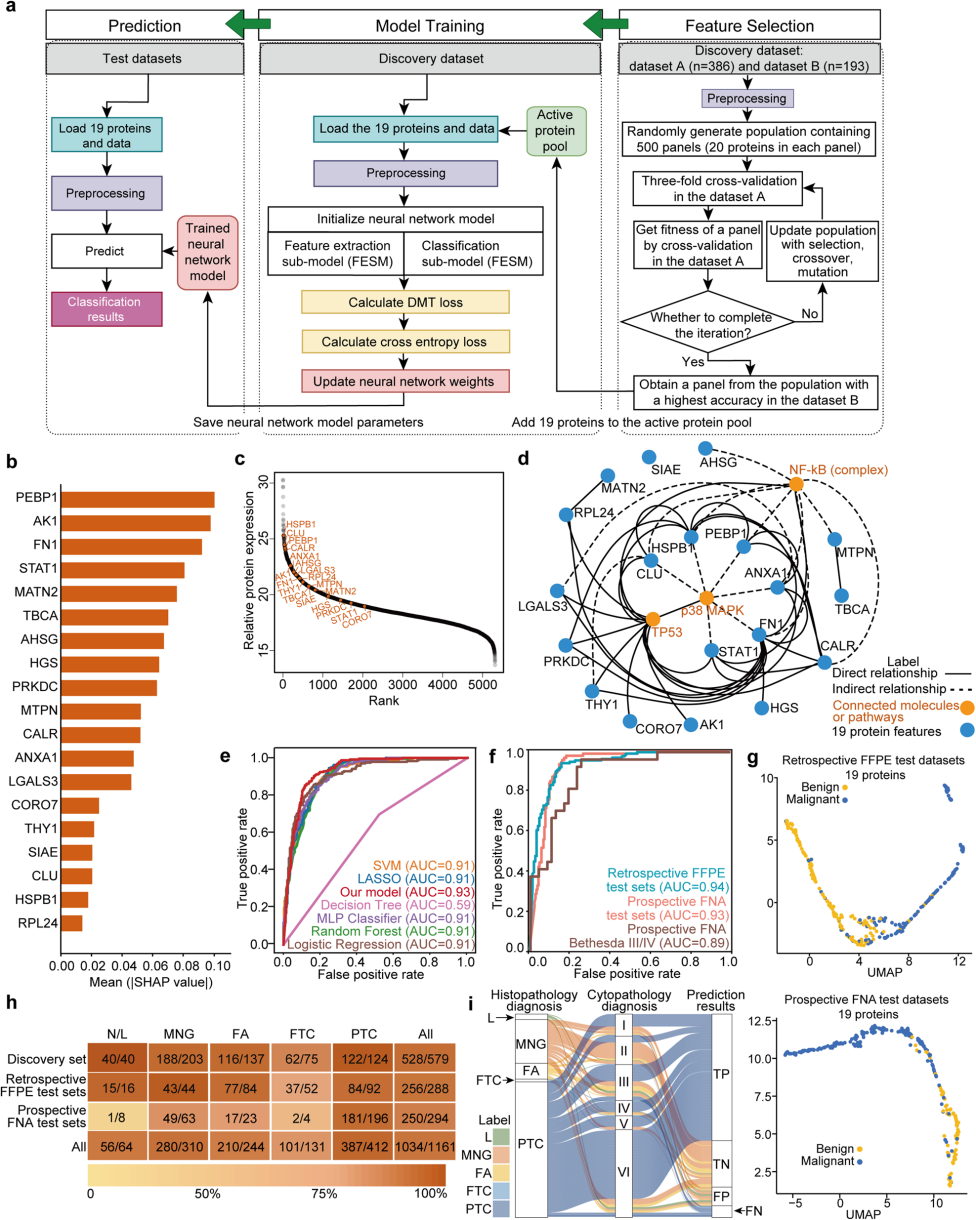
Classifier development, performance testing, and validation in independent blinded datasets. **a** Schematic workflow of the classifier development. Protein features were prioritized based on the discovery dataset. The model was trained using 19 proteins selected from the discovery dataset and further validated in test datasets. More details are described in Materials and Methods. **b** The importance rank of the selected 19 protein features was interpreted by SHapley Additive exPlanations (SHAP) algorithm. **c** Protein abundance distribution of the 19 features. **d** Network of the 19 proteins. Blue nodes and orange nodes indicate the protein features and connected molecules or pathways, respectively. Direct interactions are in solid lines and indirect interactions are in dash lines. **e** ROC plots of seven different machine learning models of 19 selected features. **f** ROC plots of the discovery set, retrospective test sets, prospective test sets and Bethesda III and IV samples in the prospective test sets. **g** UMAP plots showing the separation between benign and malignant groups in the retrospective and prospective test sets using 19 protein features with latent space. **h** Overall performance metrics of prediction of the neural network model for five specific histopathological types per set. Graduated colors in the shaded bar indicate accuracy levels. Numbers in the boxes indicate the number of correctly identified samples/total sample number. HCA and HCC were assigned as FA and FTC, respectively. **i** Sankey diagram showing the distribution ratio and correspondence between histopathology and cytopathology in the prospective sets. Histopathological type L denotes lymphocytic thyroiditis. Cytopathology scores were assigned by specialized pathologists using the Bethesda System. TP, TN, FP, and FN indicate true positive, true negative, false positive, and false negative, respectively, of the results predicted by our classifier model.

**Fig. 4:**
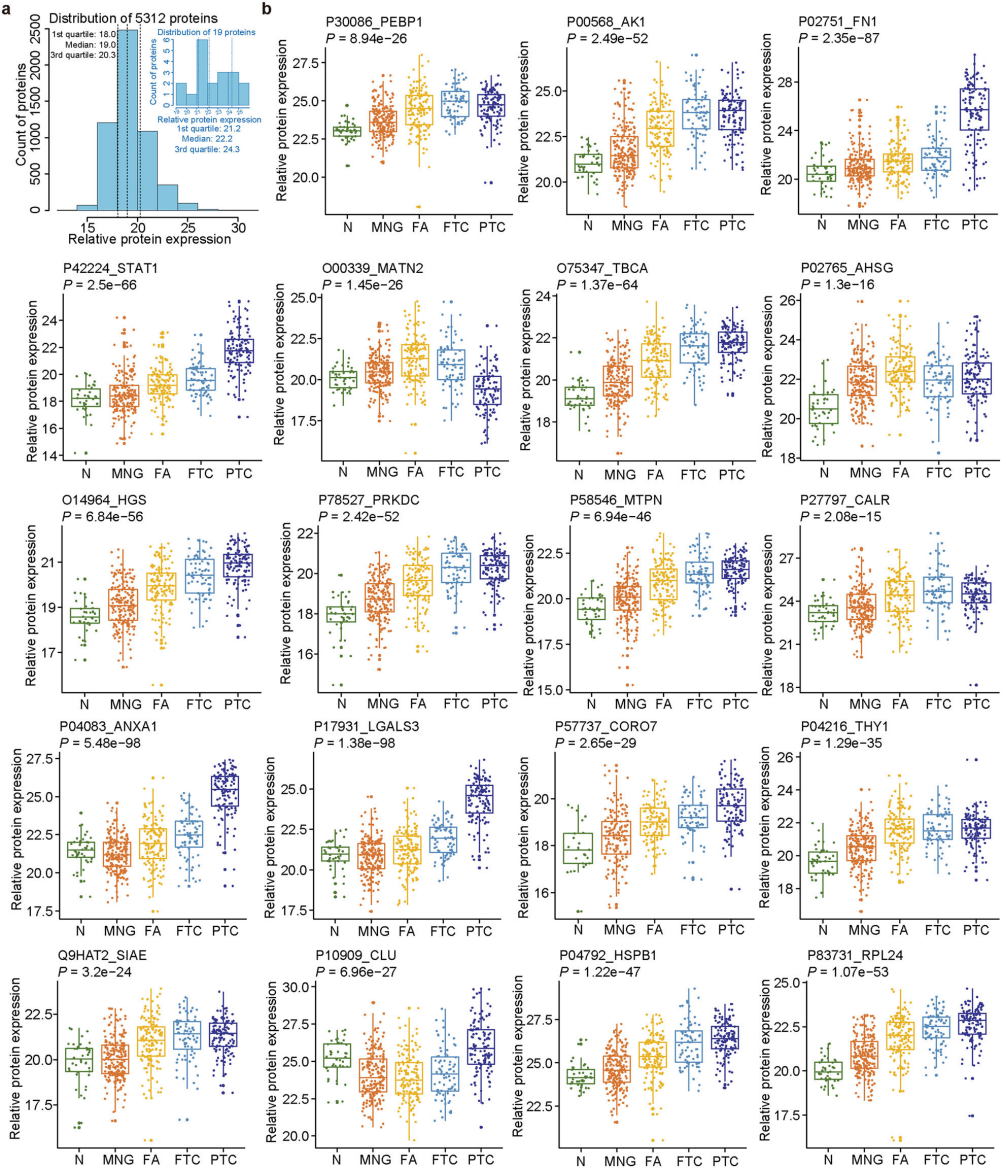
Protein expression plots for 19 selected protein features in the five histotypes of thyroid tissues in the discovery cohort. **a** The plots showing the abundance distribution of 5312 proteins and 19 selected features. **b** *y*-axis shows log_2_ values of protein expression intensity, and *x*-axis indicates tissue types. *P*-value was calculated by one-way ANOVA.

To derive a protein-based signature differentiating benign from malignant thyroid nodules, we developed a feature selection process combined with a neural network classifier based on the discovery dataset of 579 samples (Fig. 3a). Here we limited the number of selected features to no more than twenty, so that they may be readily measured as a panel by targeted proteomics in clinical laboratories. Briefly, the discovery dataset was randomly divided into dataset A containing 2/3 of the samples (*n* = 386), while the remaining samples constituted dataset B (*n* = 193) (Supplementary Fig. S4a). Protein features were selected from dataset A by a genetic algorithm^20^ combined with three-fold cross-validation. A panel of 19 proteins (Table 1) with the best accuracy for separating benign and malignant nodules was selected in dataset B according to the genetic algorithm (Fig. 3a). The 19 proteins function interactively as a whole based on their abundance in the model rather than in isolation as individual proteins. Next, the importance of the 19 protein features for the classifier was evaluated by SHapley Additive exPlanations (SHAP) algorithm^21,22^ (Fig. 3b). We further analyzed the abundance distribution of 5312 proteins and 19 selected features as shown in Figs. 3c and 4a. The quartiles of abundance distribution of 5312 proteins were 18.0 (first quartile), 19.0 (second quartile/median), and 20.3 (third quartile). The 19 protein features were higher in abundance than the median abundance of 5312 proteins, which are easier to be measured. The individual protein expression levels are shown in Fig. 4b.

**Table 1:** Nineteen proteins selected by genetic algorithm and previously known associations with thyroid physiology or pathology.

Uniprot ID	Gene name	Protein name	Thyroid cancer related	Thyroid function related
P04083	*ANXA1*	Annexin A1	Yes	Yes
P17931	*LGALS3*	Galectin-3	Yes	Yes
P02751	*FN1*	Fibronectin (FN)	Yes	Yes
P10909	*CLU*	Clusterin	Yes	Yes
P00568	*AK1*	Adenylate kinase isoenzyme 1 (AK1)	Yes	Yes
P42224	*STAT1*	Signal transducer and activator of transcription 1-alpha/beta	Yes	Yes
P30086	*PEBP1*	Phosphatidylethanolamine-binding protein 1	Yes	Yes
P27797	*CALR*	Calreticulin	Yes	Yes
P78527	*PRKDC*	DNA-dependent protein kinase catalytic subunit	Yes	Yes
O00339	*MATN2*	Matrilin-2	Yes	–
P02765	*AHSG*	Alpha-2-HS-glycoprotein	Yes	–
P04792	*HSPB1*	Heat shock protein beta-1	Yes	–
O75347	*TBCA*	Tubulin-specific chaperone A	–	Yes
P04216	*THY1*	Thy-1 membrane glycoprotein	–	Yes
Q9HAT2	*SIAE*	Sialate *O*-acetylesterase	–	–
O14964	*HGS*	Hepatocyte growth factor-regulated tyrosine kinase substrate	–	–
P58546	*MTPN*	Myotrophin	–	–
P83731	*RPL24*	60 S ribosomal protein L24	–	–
P57737	*CORO7*	Coronin-7	–	–

Twelve of the selected proteins were previously reported as relevant for thyroid cancers (Table 1), namely, alpha 2-HS glycoprotein (AHSG)^23^, annexin A1 (ANXA1)^24^, clusterin (CLU)^25^, galectin-3 (LGALS3)^26^, calreticulin (CALR)^27^, phosphatidylethanolamine-binding protein 1 (PEBP1)^28^, heat shock protein beta-1 (HSPB1)^29^, adenylate kinase isoenzyme 1 (AK1)^30^, signal transducer and activator of transcription 1-alpha/beta (STAT1)^31^, matrilin-2 (MATN2)^32^, DNA-dependent protein kinase catalytic subunit (PRKDC)^33^, and fibronectin (FN1)^34^. A further two proteins in this panel are known to be involved in various thyroid functions (Table 1), namely, tubulin folding cofactor A (TBCA)^35^, and Thy-1 membrane glycoprotein (THY1)^36^. No previous association with thyroid disease has been reported for the remaining five proteins: sialic acid acetylesterase (SIAE), hepatocyte growth factor-regulated tyrosine kinase substrate (HGS), Myotrophin (MTPN), 60S ribosomal protein L24 (RPL24), and Coronin-7 (CORO7). Although these five proteins have not yet been studied in the thyroid, we found four proteins (HGS, MTPN, RPL24, and CORO7) were directly or indirectly connected with the known thyroid cancer-related proteins by the network analysis (Fig. 3d), which indicates that the feature selection by genetic algorithm has the potential to unearth the hidden essential proteins to classify thyroid nodules. Moreover, both pathway enrichment (Supplementary Fig. S4b) and network analyses (Fig. 3d) point to the same biological process, p38 mitogen-activated protein kinase (MAPK) signaling cascade, which is predominantly activated in thyroid tumorigenesis^37^.

We next trained a neural network model designed by a multilayer perceptron (MLP) structure and loss functions. The model comprised a ‘feature extraction sub-model’ which extracts and maps features from protein data into a feature vector in latent space, and a ‘classification sub-model’ which assigns a score (from 1 to 0; nodule with score > 0.5 would be regarded as benign tissue) to the feature vector indicating the likelihood of malignancy for each sample. We designed the cross-entropy loss function by giving different weights to two parameters to deal with the imbalanced data. Details of the neural network model are described in Materials and Methods and Supplementary Notes. Since a higher specificity is required to lower the over-diagnosis rate of thyroid nodules which is the current clinical challenge in the diagnosis of thyroid nodules, we attempted to maximize specificity while maintaining sensitivity > 80% in our model optimization. We compared six alternative machine learning models with our established classification model using the 19 selected proteins. To fairly compare the performance of different algorithms, the selected protein panel was optimized using multilayer neural network within the genetic algorithm, independently from any other classifier including our designed classifier. Receiver operating characteristics (ROC) plots showed our model described here achieved the highest area under the curve (AUC) value of 0.93 in the combined test sets (Fig. 3e). Using the 19 protein features in our established neural network model with five-fold cross-validation, each specimen was re-classified into benign or malignant in the 463 randomly selected samples (80% of the discovery set) used as the training set from the discovery cohort. We validated this model on the remaining 116 samples (20% of the discovery set) from the same cohort (Fig. 3a; Supplementary Fig. S4c). Our model achieved an AUC value of 0.94 for the cross-validation sets (*n* = 579). UMAP plots of the feature latent space showed a clear separation between malignant and benign tissues using the 19-protein panel (Fig. 3g). FTC was the sample type located in the middle of the transition zone, making it the hardest histotype to predict (Supplementary Fig. S4d).

### Performance of the protein classifier

To validate this 19-protein model in independent cohorts, we first analyzed 288 pathologist-reviewed FFPE tissues (*n* = 271 patients) from three high-volume hospitals, comprising 144 benign and 144 malignant tissue samples. To ensure rigorous validation, the diagnoses were blinded during data acquisition and analyses. Each sample was analyzed using the PCT-DIA workflow in technical duplicates. Analysis of the 576 DIA maps thus generated identified 59,077 peptides, 6202 protein groups, and 6152 proteotypic proteins (Supplementary Table S2). The overall ROC plot for these retrospective independent test sets using the 19-protein model showed an AUC of 0.94 (Fig. 3f) and an accuracy of 89%. Both scatter and UMAP plots demonstrated distinct separation between benign and malignant thyroid tissues (Fig. 3g; Supplementary Fig. S4d). The overall sensitivity and specificity were 84% and 94%, respectively, with negative- (NPV) and positive-predictive values (PPV) of 85% and 93%, respectively. Further details are provided in Supplementary Tables S3 and S4.

Given that the eventual objective is to develop this protein panel for clinical application as a predictive biomarker in FNA biopsies before surgery, we extended the validation to an independent prospective patient cohort comprising 294 FNA samples from 284 patients in nine clinical centers all of whom underwent thyroid excision surgery after pre-operative FNA. The latter criterion was to ensure that histopathologic classification (as ground truth) was available for each sample. Remarkably, even from these minute amounts of FNA biopsy, we were able to generate a high-quality protein matrix containing 6210 proteotypic proteins using PCT-DIA technology (Supplementary Table S2). Using histopathological diagnoses of excised thyroid tissues as the benchmark, our model achieved an AUC value of 0.93 (Fig. 3f) and correctly identified 250 of 294 samples with 85% accuracy; and with sensitivity, specificity, PPV, and NPV of 92%, 71%, 80%, and 87%, respectively (Supplementary Table S4). The high proportion of malignant nodules was due to the fact that we only included operative nodules in our analysis. Should we use the prevalence of 30%, our model would achieve an NPV of 95%. Detailed performance metrics for each set are summarized in Fig. 3h and Supplementary Fig. S4e.

We further evaluated our classifier with the Bethesda categories of FNA samples. For indeterminate thyroid nodules (Bethesda III and IV), the AUC value of our classifier was 0.89 (Fig. 3f); 59 of 74 FNA biopsies were correctly identified with sensitivity, specificity, PPV, and NPV of 85%, 70%, 73%, and 83%, respectively, with malignant tissue prevalence of 64% (Supplementary Table S4). The distribution of each thyroid cytopathology category in the Bethesda System and our classification results are shown in a Sankey plot (Fig. 3i). Using pathological examination of surgically resected thyroid as the ground truth, cytopathologists achieved 82% overall diagnostic accuracy of FNA samples in Bethesda II, V and VI categories, while our model achieved 88% accuracy for these same nodules. These results indicate the feasibility of using MS and a machine learning-based protein classifier for tissue diagnosis.

We also assessed the classifier on different sizes of nodules and specific histopathological types of tumors. The classifier showed a more accurate prediction in nodules ≥ 1 cm in size (87.7%) than those < 1 cm (75.8%) in the prospective sets, which may be due to inaccurate sampling of small nodules. Nodules with marked lymphocytic infiltration were difficult to distinguish from malignant nodules. Ten of 44 wrongly identified nodules were thyroiditis, i.e., Hashimoto’s disease. Twenty-nine nodules were annotated with lymphocytic infiltration and only 19 were correctly identified, from which only one of seven pure lymphocytic thyroiditis samples was correctly classified as benign in the prospective sets (Fig. 3h). This may be because the histopathological changes in our present datasets are mainly present in malignant tumors. The similarly low number of samples in the training set may also have militated against the diagnostic accuracy of lymphocytic thyroiditis. The predictive accuracies for tissue histotypes were 90% for MNG and 94% for PTC in all sets (Fig. 3h). The highest accuracy was achieved for PTC, the most common thyroid malignancy accounting for ~85% of all thyroid cancers^37^. Furthermore, in a deeper dive into the model’s capability to classify the five subtypes of follicular-pattern tumors (which continue to be a challenge in clinical practice), the classifier achieved accuracies of 86%, 76%, 83%, 87%, and 87% in FA, FTC, Hürthle cell adenoma (HCA), Hürthle cell carcinoma (HCC) and follicular variant PTC (fvPTC), respectively (Supplementary Fig. S4e). The lower predictive accuracy for FTC may be ascribed to its much lower prevalence compared to PTC and, consequently, the smaller number of clinical samples analyzed. It may also reflect known similarities in histopathology and potential biological overlap between FTC and FA. While oncocytic follicular tumors were well classified by our model, the limited number of these samples in our study necessitates further validation of our model on this tumor subtype.

### Biological insights into thyroid tumor subtypes

**Fig. 5:**
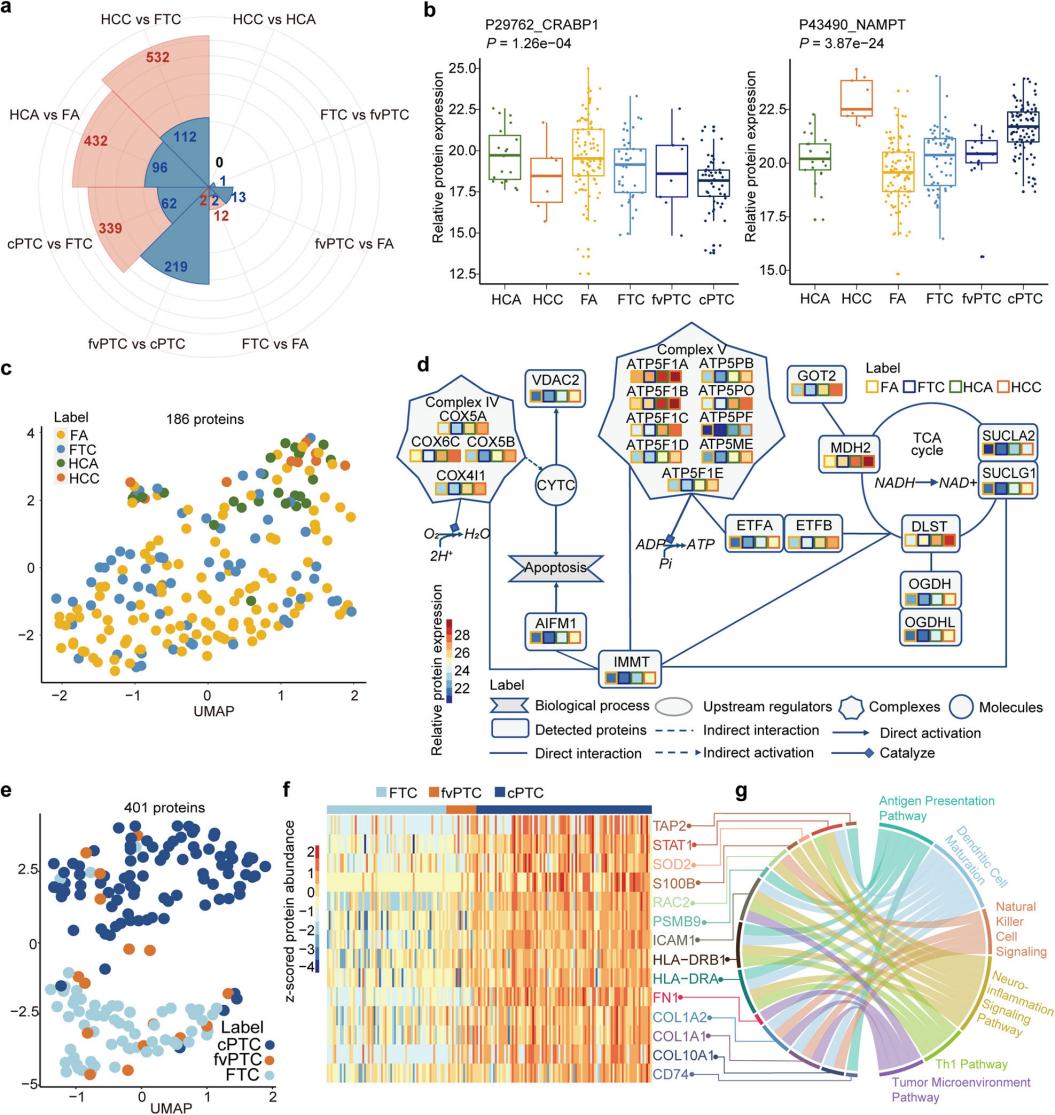
Biological insights of thyroid tumor subtypes based on proteotypic data. **a** Rose chart plotting the DEP counts of corresponding pairwise comparison for follicular-pattern tumors and control samples (cPTC). The threshold that we used was fold change > 4 and adjusted *P*-value < 0.01. The pink and blue colors represent counts of upregulated and downregulated proteins in the Rose chart, respectively. **b** Box plots showing CRABP1 and NAMPT dysregulated in six histological tumor subtypes, especially between FTC and FA. *P*-values were calculated by one-way ANOVA for six-group comparison in the box plots. **c** UMAP plot for 186 proteins distinguishing Hürthle cell tumors from other follicular neoplasms. **d** Network map showing expression of key mitochondrial proteins implicated in Hürthle cell neoplasms. **e** UMAP plot for 401 proteins distinguishing FTC from cPTC, with fvPTC as an intermediate phenotype. **f**, **g** Heatmap showing DEPs (**f**) in FTC compared with fvPTC and cPTC, with pathways (**g**) indicated in the chord plot.

Next, we asked whether the proteomic data could be used to reveal biological insights into follicular subtypes of thyroid neoplasms. We conducted eight pairwise comparisons among the follicular tumors and the control subtype of classical PTC (cPTC). Pathological differences between tissues were evaluated by the number of differentially expressed proteins (DEPs) between the various tissue types as shown in the Rose chart (Fig. 5a). In the pairwise comparison, we observed that a greater difference in histology was associated with the higher number of DEPs, further confirming that the thus acquired proteotype reflects phenotype. There were only 14 DEPs between FTC and FA, while no DEP was detected between HCC and HCA, suggesting these pairs have similar morphology. Indeed, the histological distinction between these two pairs is also a clinical challenge. We plotted the expression abundance of two DEPs, cellular retinoic acid-binding protein 1 (CRABP1) and nicotinamide phosphoribosyltransferase (NAMPT). The expression of both was different among the six tumor subtypes (Fig. 5b). Expression of CRABP1 in our dataset was higher in FA than FTC, concordant with IHC validation by other investigators^38^.

In recent years, differentiated thyroid cancers have been further sub-classified based on specific morphological features or their expected clinical course. HCA and HCC are deemed as distinct entities, with the latter demonstrating a higher propensity for vascular invasion and metastasis^39,40^. The proteomic data of this study showed these to be well-resolved from other neoplasms with hundreds of DEPs (Fig. 5a), even from the closely related FA and FTC subtypes (Fig. 5c; Supplementary Fig. S5a). Hürthle cell tumors are known for their oncocytic morphology and increased glucose uptake in fluorodeoxyglucose (FDG)-positron emission tomography scans^41^. Indeed, our data showed that 160 of 186 proteins substantially elevated (fold change > 4 and adjusted *P*-value < 0.01, Supplementary Fig. S5b) were mitochondrial proteins participating in multiple metabolic processes including the tricarboxylic acid cycle and oxidative phosphorylation (Fig. 5d; Supplementary Fig. S5c, d). These proteomic data implicate the likely biochemical processes contributing to the elevated metabolism in these tumors. Compared to the other four complexes in the oxidative phosphorylation pathway, the most strongly upregulated proteins were in complex V, which catalyzes adenosine triphosphate synthesis and potentially enhances tumor growth.

fvPTC is a subtype with mixed morphology, and we therefore examined specific differences between FTC, cPTC, and fvPTC. There were no significant proteotypic differences between FTC and fvPTC (Fig. 5a); only one DEP was identified. However, 221 proteins were differentially regulated in fvPTC compared to cPTC (Fig. 5a). Our proteotypic data showed that fvPTC overlapped with both FTC and cPTC, but resembled FTC more closely, indicating that fvPTC is potentially an intermediate entity between FTC and cPTC (Fig. 5e). This is consistent with genomic classifiers, suggesting that FTC and fvPTC share common alterations, including those in the RAS pathway^42^. Compared to FTC, the 339 proteins upregulated in cPTC (Fig. 5a) were mapped to immune-related pathways, suggesting that inflammation is involved in the pathogenesis of cPTC, which has been associated with lymphocytic thyroiditis (Fig. 5f, g).

## Discussion

Molecular diagnostics for thyroid nodules has been enabled by genomic tests due to the feasibility of analyzing small clinical samples and the increasing affordability of next-generation sequencing, however, it has also been limited to nucleic acid-based testing thus far. Several nucleic acid-based tests are commercially available through central-lab testing; their performance in clinical experience is suboptimal in terms of specificity, especially in malignancies with a low mutational burden as rigorously examined by Wang and Sosa^10^ and summarized above. Since proteins are more stable than RNA in biopsy tissue samples^17,43^, and are directly involved in cellular processes that determine tissue phenotypes^44^, we posit that protein panels such as ours can be developed as potential point-of-care diagnostic tests through widely available techniques, such as targeted mass spectrometry and/or immunohistochemistry, as complements to nucleic acid-based testing. Our study is the first step in demonstrating feasibility. The FFPE-PCT-DIA methodology used here was able to derive protein abundance data of 6749 protein groups in 1161 nodules, generating 2650 DIA proteome datasets, including replicates. Technically, our study analyzed a much larger sample size and obtained deeper proteomic coverage compared to previous publications^45–47^. Our pipeline has generated the first repository of in-depth proteome data on various thyroid pathologies. This enabled neural network analysis to mine large proteomic datasets for protein biomarkers of thyroid cancers. A panel of 19 proteins differentiated benign from malignant disease with diagnostic accuracy 85% and AUC 0.93 in prospective FNA-derived test sets and AUC of 0.89 for Bethesda III/IV in prospective sets. The fact that 14 of these 19 proteins have been implicated previously in thyroid physiology or pathology provides orthogonal validation for the inclusion of these proteins in our classifier. Analytical metrics from our proteomics data exhibited a high degree of sensitivity and specificity as shown in Supplementary Table S4. Notably, our approach works for small tissue samples obtained from FNA biopsies, making it more broadly applicable to standard clinical practice, overcoming some of the issues with RNA-based assays due to the fragility of RNA integrity.

Expanding this robust workflow to other carefully curated clinical cohorts may offer unprecedented opportunities to gain fundamental insights into the molecular pathogenesis of diseases and address critical unmet clinical needs beyond thyroid cancer. Although this classifier has been retrospectively and prospectively validated in several independent clinical centers, further validation performed on FNA biopsies of larger prospective cohorts comprising indeterminate nodules (Bethesda III/IV) is required to support the utility of this approach in standard clinical practice.

Despite the high accuracy in distinguishing benign from malignant thyroid nodules, the major limitation for most algorithms is distinguishing FTC from FA. Indeed, the proteotype data presented here even suggest that follicular neoplasms may represent a disease continuum, in which differences exist at the extremes of phenotypes, but with significant overlap in-between. Alternatively, some of the nodules classified as benign adenomas may simply represent pre-malignant stages diagnosed prior to the overt capsular or vascular invasion, even though pre-requisite conditions for the invasion were already present. The proteomic difference between benign and malignant follicular tumors appeared subtle, therefore larger sample size is required to make a distinction. Future studies should also focus on the specific distinction between FA and FTC.

Our present study is a proof of principle to show that DIA-based classifiers can be used to classify thyroid nodules. Targeted assays should be developed in the future to implement real-world applications. The protein classifier has a higher specificity, but a slightly lower sensitivity compared to counterpart genomic tests, serving as a complement to genomic tests.

Artificial intelligence has enabled significant progress in the field of image processing for thyroid nodule evaluation^48^. It is likely that combining a biomarker protein panel with image-based evaluation and genotype data could refine and enhance diagnostic assessments of indeterminate thyroid nodules to reduce the costs and morbidity of over-treatment, although the integration of these multi-dimensional and multi-modality datasets may be challenging and create some redundancy between some of these techniques.

In conclusion, we present the first protein-based neural network classifier for thyroid nodules. This large-scale thyroid proteome profile of 1161 thyroid nodules coupled with a neural network model demonstrates for the first time, the power of a protein-based disease classifier with the potential for rapid translation into clinical practice to complement conventional cytopathology.

## Materials and methods

### Patients and tissue samples

We initially collected 581 thyroid nodule samples. After the pathologist review of all sections to confirm tissue diagnosis, the discovery sample set of 579 thyroid nodules from 578 patients comprised FA (*n* = 137), MNG (*n* = 203), PTC (*n* = 124) and FTC (*n* = 75) from the Singapore General Hospital. Normal thyroid tissues (N, *n* = 40) were taken from cases of laryngectomy or pharyngo-laryngo-esophagectomy, in which the thyroid gland was surgically removed incidental to radical surgery for non-thyroid cancers. These patients had no history of thyroid disease, prior chemotherapy or radiation.

Hematoxylin and eosin-stained slides from tissue blocks of each patient were reviewed by an experienced histopathologist who marked out the disease region for tissue coring. Tissue cores (1 mm diameter, 0.5–1 mm thick, approximate weight 0.6–1.2 mg, including wax) were punched from the pathological areas of interest in blocks of FFPE thyroid tissues. Based on the assessment of an experienced pathologist for each punch, a region of interest was comprised of ~100% cancer cells. Three adjacent tissue cores from the same region were made for each case as biological replicates in the discovery set. These thyroid tissues were obtained from four clinical centers in Singapore and China spanning 2011–2019, with the ethics approval of each hospital.

We analyzed a total of 288 FFPE tissue cores from 271 patients obtained in three hospitals as multi-center blinded retrospective test sets. These samples were classified into 16 N, 44 MNG, 84 FA, 52 FTC, and 92 PTC cases using the same histology classification system and sampling method as the Singapore samples based on the standardized World Health Organization classification^18^. A single core was made from each case.

Furthermore, we prospectively collected 395 FNA biopsies from nine clinical centers, of which 294 nodules were surgically removed. Prospective validation was performed on the 294 FNA biopsies from 284 patients treated in different hospitals in China and Singapore. Each patient proceeded to thyroid surgery after FNA. Definitive histopathological diagnosis of surgically excised thyroid tissue in each case was determined. This series comprised 8 lymphocytic thyroiditis (L), 63 MNG, 23 FA, 4 FTC, and 196 PTC (*n* = 294). All FNA samples were categorized according to the Bethesda System for Reporting Thyroid Cytopathology. Histological and cytopathological diagnoses of these samples were blinded during the entire workflow of prospective sample processing, mass spectrometry analysis, and predictive data analysis.

The study methodologies conformed to the standards set by the Declaration of Helsinki and were approved by the local ethics committee. The experiments were undertaken with the understanding and written consent of each subject.

### Batch design

To minimize batch effects among different lots of analyzed samples, 1803 thyroid FFPE cores from 581 thyroid nodules with three biological replicates (581 × 3) and 60 technical replicates were randomly allocated into 121 discovery batches to minimize the batch effect for this large-scale sample preparation (Supplementary Fig. S1a). Batch 121 in the discovery dataset had only 3 samples. 60 technical replicates were analyzed independently from the DIA-MS analysis. Each batch contained 15 thyroid samples, one mouse liver sample as QC for PCT, and one thyroid pooled sample containing all five types of thyroid tissues for MS. The technical replicates were distributed randomly as one of the 15 samples per batch. In this discovery phase analysis, tissue cores were divided into multiple batches with balanced histopathology diagnoses in each batch.

In the external validation phase analysis, 288 FFPE cores were analyzed in technical duplicates for a total of 576 MS runs in 39 batches for retrospective test sets and 395 fine needle biopsies in 27 batches for prospective test sets (Supplementary Fig. S1b).

### Dewaxing, rehydration, and hydrolysis of FFPE tissues

For each case in the discovery sample set, three biological replicates of FFPE tissue cores were processed. Sample weights were recorded before dewaxing in heptane (Sigma-Aldrich) and successive rehydration in 100% ethanol (Sigma-Aldrich), 90% ethanol, 75% ethanol at room temperature. Formic acid (0.1%) (Sigma-Aldrich) was added next to achieve C–O hydrolysis of protein methylol products and then washed with 100 mM Tris-HCl (pH 10, Sigma-Aldrich) to establish conditions for base hydrolysis at 95 °C. The sample was then snap cooled to 4 °C. Twelve samples were lost after dewaxing.

### Tissue lysis, protein extraction, and protein digestion

The red blood cells (RBCs) in FNA samples were firstly removed by 500 μL ACK lysis buffer (Solarbo, Chian) and then centrifuged at 450× *g* for 10 min to collect the precipitated content. Dewaxed FFPE samples and RBC-depleted FNA biopsies were lysed in 6 M urea (Sigma-Aldrich) and 2 M thiourea (Sigma-Aldrich) using PCT programmed for 90 cycles of 25 s at 45,000 p.s.i. and 10 s at ambient pressure and 30 °C. After lysis, 10 mM Tris(2-carboxyethyl)phosphine hydrochloride (Sigma-Aldrich) and 40 mM iodoacetamide (Sigma-Aldrich) were simultaneously added to the solution and incubated in the dark with gentle vertexing for 30 min, after which LysC (Hualishi Tech. Ltd., Beijing, China) was added at a ratio of 40:1 (protein to LysC). PCT-assisted LysC digestion was performed with the following setting: 45 cycles of 50 s at 20,000 p.s.i. and 10 s at ambient pressure and 30 °C. Final tryptic digestion was performed at a ratio of 50:1 (protein to trypsin (Hualishi Tech. Ltd., Beijing, China)) by PCT with the following setting: 90 cycles of 50 s at 20,000 p.s.i. and 10 s at ambient pressure and 30 °C. Peptides were desalted before LC-MS analysis.

### DIA-MS data analysis

Peptides were separated using Ultimate 3000 or nanoLC-MS/MS system (DIONEX UltiMate 3000 RSLCnano System, Thermo Fisher Scientific™, San Jose, USA) equipped with 15 cm × 75 μm ID fused silica column custom packed with 1.9 μm 120 Å C18 aqua. To increase the throughput of sample detection, we chose a shorter LC gradient of 45 min (68 min inject-to-inject). Peptides were separated at 300 nL/min in a 3%–25% linear gradient of buffer B (buffer A: 2% acetonitrile, 0.1% formic acid; buffer B: 98% acetonitrile, 0.1% formic acid). Peptides eluted from analytical columns were ionized at a potential +2.0 kV into Q Exactive HF mass spectrometer (Thermo Fisher Scientific™, San Jose, USA). A full MS scan was acquired analyzing 390–1010 *m/z* at a 60,000 resolution (at *m/z* 200) in the Orbitrap using an AGC target value of 3e6 charges and the maximum injection time of 100 ms. After the full MS scan, 24 MS/MS scans were acquired, each with a 30,000 resolution (at *m/z* 200), AGC target value of 1e6 charges, normalized collision energy of 27%, with the default charge state set to 2, maximum injection time set to auto. The cycle of 24 MS/MS scans (center of isolation window) with three kinds of wide isolation window was as follows (*m/z*): 410, 430, 450, 470, 490, 510, 530, 550, 570, 590, 610, 630, 650, 670, 690, 710, 730, 770, 790, 820, 860, 910, 970. The entire MS and MS/MS scan acquisition cycle took ~3 s and was repeated throughout the LC/MS analysis.

We acquired a total of 2650 effective DIA files that could be analyzed further. Specifically, these consisted of 1780 files from the discovery dataset; 576 files from the retrospective test dataset (288 samples × 2 technical replicates); 294 files (no replicates) from the prospective test datasets.

In the discovery set, 581 nodules × 3 biological replicates were first obtained, of which 13 samples were lost because insufficient peptide mass was extracted for acquiring all replicates; additionally 60 technical replicates were added, 4 of which were lost. Furthermore, during slide review by pathologists and sample preparation, 6 samples were excluded due to unmatched histological tissue type. Thus, there was a total of (581 × 3 + 60) – 13 – 4 – 6 = 1780 DIA files.

In the prospective set, 101 of 395 nodules were not excised due to lack of definite histopathological diagnoses, and then they were excluded. After filtering, 294 DIA files for prospective validation were analyzed.

DIA raw files were analyzed using DIA-NN (v1.7.15)^49^ and against our previously released thyroid-specific spectral library. The cysteine carbamidomethylation was set as a fixed modification, while the methionine oxidation was as a variable modification. Peptide length range, precursor *m/z* range, and fragment ion *m/z* range were set as 6–30, 300–1500, and 100–1800, respectively. 1% false discovery rate (FDR) of the precursor was applied. Precursor IDs that were likely to be caused by interferences were removed. Other parameters were used by default. The protein matrix that we used for downstream analyses was the abundance average of replicates from the same thyroid tissue regardless of biological or technical replicates.

### Data quality control

We first assessed data quality by analyzing control samples. The QC samples in each batch were mouse liver samples (PCT-QC) and pooled thyroid samples (DIA-QC). Additional QC samples were analyzed as technical replicates for MS. Biological replicates were also analyzed to determine the extent of heterogeneity of thyroid diseases. Reproducibility of spiked-in mouse liver samples and thyroid pooled samples showed that PCT and MS instruments were stable during data acquisition (Supplementary Fig. S2a, b), with a median coefficient of variance (CV) < 0.04. MS data of 56 randomly selected paired thyroid samples in the discovery cohort and 288 samples × 2 technical replicates in the retrospective set had a median Spearman correlation coefficient of 0.91 and 0.97, respectively (Supplementary Fig. S2c). CV for proteins in technical replicates was 0.02; and that in biological replicates was 0.04, slightly higher than that in technical replicates indicating minimal tissue heterogeneity in the biology of thyroid disease (Supplementary Fig. S2d). Finally, we compared the Spearman correlation of technical replicates and biological replicates for the 56 samples (Supplementary Fig. S2e). The correlation of biological replicates was lower than that of technical replicates, probably reflecting tissue heterogeneity. For the protein identification in the three datasets, there were 5957 proteins identified and quantified in all the three sets (Supplementary Fig. S2f).

### Protein data preprocessing

Datasets (discovery dataset, retrospective test datasets, and prospective test datasets) from twelve clinical centers were used to develop and validate the neural network model. Considering that most missing values occurred when the protein content was below the detection threshold, imputation was performed by filling in all the missing values with [*D*_*min*_], where *D*_*min*_ was the minimum of all available feature values in the discovery set, and [·] is the ceiling operator. The minimum value was 12 for the discovery set and all the test sets used. The missing values of all the datasets, which account for 51% of all data, were imputed with this value.

After the imputation step, for each feature, the mean and variance of the feature were estimated from the discovery set, and each feature of every training sample was normalized as

1 $$D^n = \frac{{D - \mu }}{\sigma }$$

Obtained *μ* and *σ* were estimated from the discovery dataset and then applied for corresponding protein features in the retrospective and prospective test datasets. Python’s ‘pandas’ library was used for data preprocessing.

### Development of neural network classifier

An artificial neural network was developed to classify a sample (a vector of selected protein features) into one of the two classes, namely benign (B) or malignant (M). This was done in the three stages:

1. Protein feature selection using a genetic algorithm;
2. Neural network training;
3. Sample classification using the trained neural network.

The following explains the three modules and the pipeline.

### Stage 1: Feature selection

The feature selection consisted of two steps (see Supplementary Fig. S6a). The first step was initial feature screening based on available information. Of the initial 6689 protein features, 1302 were selected from the differentially expressed proteins of benign and malignant samples in the discovery dataset, the published literature on thyroid or thyroid cancer, and from the favorable or unfavorable prognosis of thyroid cancer annotated by TCGA or OMIM databases.

As the second step, the genetic algorithm^20^ was used to select an optimal combination of 19 proteins from the initial 767 ones with missing rates < 35% of samples. The evaluation of feature missing rates and feature counts was described in the Supplementary Notes. Python’s deap library was adopted here for genetic algorithm-based feature selection. In the genetic algorithm, evolutionary operations — crossover, mutation, and selection operations were used to generate new protein feature combinations from existing protein feature combinations. The genetic algorithm eliminated low fitness combinations at every iteration and generates new combinations based on the remaining high fitness combinations.

The discovery set was divided into dataset A containing 386 samples (2/3 of the discovery set) for cross-validation and dataset B containing 193 samples (1/3 of the discovery set) for validation. Dataset A was used to calculate the fitness of individuals during the genetic algorithm iteration, while dataset B was used to evaluate the performance of each combination. A fitness value was calculated for each candidate combination solution in dataset A. For combination solution *C*, the fitness value was defined as

2 $$F^{{{\mathrm{C}}}} = \frac{1}{3}\mathop {\sum }\limits_{k = 1}^3 A_k^{{{\mathrm{C}}}}$$

where $A_k^C$ was the accuracy of the 3-fold cross-validation^50^, which was computed from the difference between the output of the classifier and the true label. 1.7% of data was imputed for the newly selected 19 protein features.

We also compared the different feature selection methods with genetic algorithm and evaluated the stability of the selected features which were described in the Supplementary Notes in detail.

### Stage 2: Neural network model training

The neural network classifier was a nonlinear function that takes a vector of 19 selected protein features as the input and outputs a class label of either 1 (for benign) or 0 (for malignant). This module consisted of the following three steps: (1) model structure design; (2) manifold learning-based loss function design; and (3) model training.

An MLP structure was chosen for the neural network, shown in Supplementary Fig. S6b. The MLP model consists of a feature extraction sub-model and a classification sub-model, trained in an end-to-end fashion. The feature extraction sub-model extracts effective feature vectors (*V*_*i*_), and the classification sub-model performs diagnostic classification (*Y*_*i*_) based on the classification information.

A manifold learning-based method, deep manifold transformation (DMT)^51^, was applied for feature representation learning whereas the commonly used cross-entropy was used to constrain the supervised classification. The total loss function is defined as

3 $$L = L_{DMT} + \alpha _1L_e + \alpha _2L_r$$

where *L*_*DMT*_ was a cross-layer constraint that preserves manifold structure between the input and the latent feature layers, *L*_*e*_ was a cross-entropy loss for classification, *L*_*r*_ was an L2 regularization loss for reducing overfitting, and *α*_1_, *α*_2_ > 0 were the weights. The three loss terms are defined below.

The DMT loss was defined by a cross-layer two-way divergence or fuzzy set information for discriminant^52^

4 $$L_{DMT}=\mathop{\sum }\limits_{\begin{array}{*{20}{c}} {i \ne j,} \\ {i,j \in \left\{ {1,2,3, \cdots ,N} \right\}} \end{array}} P_{ij}{{{\mathrm{log}}}}\frac{{P_{ij}}}{{Q_{ij}}} + (1 - P_{ij}){{{\mathrm{log}}}}\frac{{1 - P_{ij}}}{{1 - Q_{ij}}}$$

where *P*_*ij*_ was the similarity in input space between point *i* and point *j*, and *Q*_*ij*_ was the similarity in latent space between point *i* and point *j*, whose computation is described below, and *N* is the number of nodules. The similarities are calculated as follows. First, the distance matrix of input space and latent space was calculated.

$$D^X = D^{(0)} = \left\{ {D_{ij}^X = \left\| {X_i - X_j} \right\|_2,i,j \in \{ 1..N\} } \right\}$$

$$D^Z = D^{(L)} = \left\{ {D_{ij}^Z = \left\| {V_i - V_j} \right\|_2,i,j \in \{ 1..N\} } \right\}$$

where feature vectors *V*_*i*_, *V*_*j*_ were extracted from protein samples *X*_*i*_, *X*_*j*_ by our MLP model, (0) and (L) are the index of the network layer as shown in Supplementary Fig. S6b. Secondly, t-distribution’s kernel function *κ*(*D*,*v*) was used to transform the distance matrix *D* into a matrix *A*:

$$A = \kappa \left( {D,\nu } \right) = \frac{{{\it{\Gamma}} \left( {\frac{{\nu + 1}}{2}} \right)}}{{\sqrt {\nu \pi } {\it{\Gamma}} \left( {\frac{\nu }{2}} \right)}}\left( {1 + \frac{{D \circ D}}{\nu }} \right)^{ - \frac{{(\nu + 1)}}{2}}$$

where, *Γ*(·) was a gamma function. *v* was the t-distribution’s degree of freedom.

Since the similarity was asymmetric, we used the function *S*(·) for symmetrization.

5 $$S(A) = A + A^T - 2A \circ A^T$$

where ◦ was Hadamard product. Finally, we defined *P* and *Q*.

6 $$\begin{array}{*{20}{c}} {P = S\left( {2\pi \cdot \kappa \left( {D^X,\nu ^X} \right)^2} \right)} \\ {Q = S\left( {2\pi \cdot \kappa \left( {D^Z,\nu ^Z} \right)^2} \right)} \end{array}$$

We applied different parameters of freedom degrees *v* in the input space and the latent space to compensate for the differences in feature dimensions. We assumed that the distribution of the input spatial distances was normal, so that *v*^*X*^ was a sufficiently large number, *v*^*X*^ = 100. In the latent space, we used the standard t-distribution and set *v*^*Z*^ = 1.

The cross-entropy loss *L*_*e*_ was calculated as

7 $$\begin{array}{*{20}{c}} {L_e = - \mathop {\sum}\limits_{i = 1}^N {\left[ {\beta \,Y_i\log \hat Y_{{{\mathrm{i}}}} + \left( {2 - \beta } \right)\left( {1 - Y_i} \right)\log \left( {1 - \hat Y_i} \right)} \right]} } \end{array}$$

where *Y*_*i*_ was the real one-hot label of the nodule, $\hat Y_i$ was the classification vector predicted by the classification sub-model, and *β* was the penalty parameter to deal with the imbalanced data. The L2 regularizer was defined as the 2-norm of MLP weight *W* as

8 $$L_r = \left\| {{{\mathrm{W}}}} \right\|_2^2 = \mathop {\sum}\limits_{i = 1}^M {w_i^2}$$

where *M* was the number of parameters.

The MLP training was performed using the training dataset from the discovery set. Python’s PyTorch library was used for model training. We trained the model for 100 epochs.

We used 5-fold cross-validation for the training and hyperparameter determination, and then selected the model with the highest AUC in the validation set. The best set of hyperparameter values was empirically chosen to be *α*_1_ = 1 × 10^3^, *α*_2_ = 50, *β* = 1.6, learning rate = 2 × 10^−2^, batch size = 256. The best model’s AUC in the validation set was 0.951.

### Stage 3: Sample classification

The trained MLP was used as the classifier for the diagnosis of unknown samples. Given the 19 features of one sample, the model would output a classification vector $\hat Y_i = \left\{ {\hat y_{{{\mathrm{i}}}}^0,\hat y_{{{\mathrm{i}}}}^1} \right\}$, $\hat y_{{{\mathrm{i}}}}^0$ and $\hat y_{{{\mathrm{i}}}}^1$describe the probability that the sample was benign or malignant. The class prediction *Pi* was calculated as

9 $$P_i = \left\{ {\begin{array}{*{20}{c}} 1 & {if\,\hat y_{{{\mathrm{i}}}}^0 \,<\, \hat y_{{{\mathrm{i}}}}^1} \\ 0 & {if\,\hat y_{{{\mathrm{i}}}}^0 \ge \hat y_{{{\mathrm{i}}}}^1} \end{array}} \right.$$

where *P*_*i*_ = 0 means the tissue was predicted to be malignant, and *P*_*i*_ = 1 means benign.

Moreover, six alternative models were compared with our established model using the 19 proteins, which are described in the Supplementary Notes.

### Statistical analysis

Statistical analysis was performed using R software (version 3.5.1) with pheatmap, UMAP, and R package plot functions. Proteins in the heatmaps were hierarchical clustered by the method of centroid. CV was calculated as the ratio of the standard deviation to the mean. The prevalence for each cohort was based on the ratio of malignant to total tissues. Sensitivity, specificity, PPV, and NPV values were calculated following the established methodology, and each value was calculated with 95% Wilson confidence intervals^53^. Biological insights were analyzed by IPA (version 49309495). The interactions among the 19 proteins were retrieved from the IPA with default settings and displayed by Cytoscape (version 3.8.2) with the radial layout. One-way ANOVA was used to calculate *P*-values in the expression of 19 protein features.

## Supplementary information

supplementary materials

Supplementary information, Table S1

Supplementary information, Table S2

Supplementary information, Table S3

## Data Availability

All data are available in the manuscript. MS raw data were deposited in iProX (IPX0001444000). Code was deposited in Github (https://github.com/zangzelin/thyroid-project.git).
