# Author Correction: Artificial intelligence defines protein-based classification of thyroid nodules

**DOI:** 10.1038/s41421-022-00471-6

**Published:** 2022-09-30

**Authors:** Yaoting Sun, Sathiyamoorthy Selvarajan, Zelin Zang, Wei Liu, Yi Zhu, Hao Zhang, Wanyuan Chen, Hao Chen, Lu Li, Xue Cai, Huanhuan Gao, Zhicheng Wu, Yongfu Zhao, Lirong Chen, Xiaodong Teng, Sangeeta Mantoo, Tony Kiat-Hon Lim, Bhuvaneswari Hariraman, Serene Yeow, Syed Muhammad Fahmy Alkaff, Sze Sing Lee, Guan Ruan, Qiushi Zhang, Tiansheng Zhu, Yifan Hu, Zhen Dong, Weigang Ge, Qi Xiao, Weibin Wang, Guangzhi Wang, Junhong Xiao, Yi He, Zhihong Wang, Wei Sun, Yuan Qin, Jiang Zhu, Xu Zheng, Linyan Wang, Xi Zheng, Kailun Xu, Yingkuan Shao, Shu Zheng, Kexin Liu, Ruedi Aebersold, Haixia Guan, Xiaohong Wu, Dingcun Luo, Wen Tian, Stan Ziqing Li, Oi Lian Kon, Narayanan Gopalakrishna Iyer, Tiannan Guo

**Affiliations:** 1 Westlake Laboratory of Life Sciences and Biomedicine, Key Laboratory of Structural Biology of Zhejiang Province, School of Life Sciences, Westlake University, Hangzhou, Zhejiang China; 2 Institute of Basic Medical Sciences, Westlake Institute for Advanced Study, Hangzhou, Zhejiang China; 3 Research Center for Industries of the Future, Westlake University, No.18 Shilongshan Road, Hangzhou, Zhejiang China; 4 Department of Anatomical Pathology, Division of Pathology, Singapore General Hospital, Singapore, Singapore; 5 School of Engineering, Westlake University, No.18 Shilongshan Road, Hangzhou, Zhejiang China; 6 Westlake Omics (Hangzhou) Biotechnology Co., Ltd., No.1 Yunmeng Road, Hangzhou, Zhejiang China; 7 Department of Thyroid Surgery, the First Hospital of China Medical University, Shenyang, Liaoning China; 8 Cancer Center, Department of Pathology, Zhejiang Provincial People’s Hospital, Affiliated People’s Hospital, Hangzhou Medical College, Hangzhou, Zhejiang China; 9 Department of General Surgery, The Second Hospital of Dalian Medical University, Dalian, Liaoning China; 10 Department of Pathology, The Second Affiliated Hospital of College of Medicine, Zhejiang University, Hangzhou, Zhejiang China; 11 Department of Pathology, the First Affiliated Hospital, College of Medicine, Zhejiang University, Hangzhou, Zhejiang China; 12 Department of Head and Neck Surgery, National Cancer Center Singapore, Singapore, Singapore; 13 Division of Medical Sciences, National Cancer Center Singapore, Singapore, Singapore; 14 Department of Surgical Oncology, the First Affiliated Hospital, College of Medicine, Zhejiang University, Hangzhou, Zhejiang China; 15 Department of Urology, The Second Hospital of Dalian Medical University, Dalian, Liaoning China; 16 Department of Ultrasound, Sir Run Run Shaw Hospital, Zhejiang University School of Medicine, Hangzhou, Zhejiang China; 17 Liaoning Laboratory of Cancer Genetics and Epigenetics and Department of Cell Biology, College of Basic Medical Sciences, Dalian Medical University, Dalian, Liaoning China; 18 Department of Ophthalmology, The Second Affiliated Hospital, Zhejiang University School of Medicine, Hangzhou, Zhejiang China; 19 Cancer Institute (Key Laboratory of Cancer Prevention and Intervention, China National Ministry of Education, Key Laboratory of Molecular Biology in Medical Sciences, Zhejiang, China), The Second Affiliated Hospital, Zhejiang University School of Medicine, Hangzhou, Zhejiang China; 20 Department of Clinical Pharmacology, College of Pharmacy, Dalian Medical University, Dalian, Liaoning China; 21 Department of Biology, Institute of Molecular Systems Biology, ETH Zurich, Zurich, Switzerland; 22 Faculty of Science, University of Zurich, Zurich, Switzerland; 23 Department of Endocrinology, Guangdong Provincial People’s Hospital, Guangdong Academy of Medical Sciences, Guangzhou, Guangdong China; 24 Department of Endocrinology, Zhejiang Provincial People’s Hospital, Affiliated People’s Hospital, Hangzhou, Zhejiang China; 25 Department of Surgical Oncology, Affiliated Hangzhou First People’s Hospital, Zhejiang University School of Medicine, Hangzhou, Zhejiang China; 26 Department of General Surgery, PLA General Hospital, Beijing, China; 27 Westlake Laboratory of Life Sciences and Biomedicine, Westlake University, Hangzhou, Zhejiang China

**Keywords:** Proteomics, Cancer models

Correction to: *Cell Discovery* (2022) 8:85

10.1038/s41421-022-00442-x published online 06 September 2022

In this article, the authors have found an error in Data Availability section, the iProX ID is incorrect. The correct one is IPX0001444000. We are sorry for the inconvenience.

