# Supplementary material for: Artificial intelligence defines protein-based classification of thyroid nodules
Source: Cell Discov. 2022 Sep 6;8:85. doi: 10.1038/s41421-022-00442-x (PMC9448820; doi:10.1038/s41421-022-00442-x)
Supplement: Supplementary file 1 — supplementary materials [file 41421_2022_442_MOESM1_ESM.pdf]

Supplementary Materials

Artificial intelligence defines protein-based classification of thyroid nodules

Yaoting Sun[1,2,3,#], Sathiyamoorthy Selvarajan[4,#], Zelin Zang[5,#], Wei Liu[6,#], Yi Zhu[1,2,3,#], Hao Zhang[7,#], Wanyuan Chen[8], Hao Chen[6], Lu Li[1,2,3], Xue Cai[1,2,3], Huanhuan Gao[1,2,3], Zhicheng Wu[1,2,3], Yongfu Zhao[9], Lirong Chen[10], Xiaodong Teng[11], Sangeeta Mantoo[4], Tony Kiat-Hon Lim[4], Bhuvaneswari Hariraman[12], Serene Yeow[13], Syed Muhammad Fahmy Alkaff[4], Sze Sing Lee[13], Guan Ruan[6], Qiushi Zhang[6], Tiansheng Zhu[1,2,3], Yifan Hu[6], Zhen Dong[1,2,3], Weigang Ge[6], Qi Xiao[1,2,3], Weibin Wang[14], Guangzhi Wang[9], Junhong Xiao[9], Yi He[15], Zhihong Wang[7], Wei Sun[7], Yuan Qin[7], Jiang Zhu[16], Xu Zheng[17], Linyan Wang[18], Xi Zheng[19], Kailun Xu[19], Yingkuan Shao[19], Shu Zheng[19], Kexin Liu[20], Ruedi Aebersold[21,22], Haixia Guan[23], Xiaohong Wu[24], Dingcun Luo[25], Wen Tian[26], Stan Ziqing Li[5,*], Oi Lian Kon[13,*], Narayanan Gopalakrishna Iyer[12,13,*], Tiannan Guo[1,2,3] *

[1]Westlake Laboratory of Life Sciences and Biomedicine, Key Laboratory of Structural Biology of Zhejiang Province, School of Life Sciences, Westlake University, Hangzhou, Zhejiang, China;

[2]Institute of Basic Medical Sciences, Westlake Institute for Advanced Study, Hangzhou, Zhejiang, China;

[3]Research Center for Industries of the Future, Westlake University, Hangzhou, Zhejiang, China;

[4]Department of Anatomical Pathology, Division of Pathology, Singapore General Hospital, Singapore, Singapore;

[5]School of Engineering, Westlake University, Hangzhou, Zhejiang, China;

[6]Westlake Omics (Hangzhou) Biotechnology Co., Ltd., Hangzhou, Zhejiang, China;

[7]Department of Thyroid Surgery, the First Hospital of China Medical University, Shenyang, Liaoning, China;

[8]Cancer Center, Department of Pathology, Zhejiang Provincial People's Hospital, Affiliated People's Hospital, Hangzhou Medical College, Hangzhou, Zhejiang, China;

[9]Department of General Surgery, The Second Hospital of Dalian Medical University, Dalian, Liaoning, China;

[10]Department of Pathology, The Second Affiliated Hospital of College of Medicine, Zhejiang University, Hangzhou, Zhejiang, China;

[11]Department of Pathology, the First Affiliated Hospital, College of Medicine, Zhejiang University, Hangzhou, Zhejiang, China;

[12]Department of Head and Neck Surgery, National Cancer Centre Singapore, Singapore, Singapore;

[13]Division of Medical Sciences, National Cancer Centre Singapore, Singapore, Singapore;

[14]Department of Surgical Oncology, the First Affiliated Hospital, College of Medicine, Zhejiang University, Hangzhou, Zhejiang, China;

[15]Department of Urology, The Second Hospital of Dalian Medical University, Dalian, Liaoning, China;

[16]Department of Ultrasound, Sir Run Run Shaw Hospital, Zhejiang University School of Medicine, Hangzhou, Zhejiang, China;

[17]Liaoning Laboratory of Cancer Genetics and Epigenetics and Department of Cell Biology, College of Basic Medical Sciences, Dalian Medical University, Dalian, Liaoning, China;
[18]Department of Ophthalmology, The Second Affiliated Hospital, Zhejiang University School of Medicine, Hangzhou, Zhejiang, China;
[19]Cancer Institute (Key Laboratory of Cancer Prevention and Intervention, China National Ministry of Education, Key Laboratory of Molecular Biology in Medical Sciences, Zhejiang Province, China), The Second Affiliated Hospital, Zhejiang University School of Medicine, Hangzhou, Zhejiang, China;
[20]Department of Clinical Pharmacology, College of Pharmacy, Dalian Medical University, Dalian, Liaoning, China;
[21]Department of Biology, Institute of Molecular Systems Biology, ETH Zurich, Zurich, Switzerland;
[22]Faculty of Science, University of Zurich, Zurich, Switzerland;
[23]Department of Endocrinology, Guangdong Provincial People's Hospital, Guangdong Academy of Medical Sciences, Guangzhou, Guangdong, China;
[24]Department of Endocrinology, Zhejiang Provincial People’s Hospital, Affiliated People’s Hospital, Hangzhou Medical College, Hangzhou, Zhejiang, China;
[25]Department of Surgical Oncology, Affiliated Hangzhou First People's Hospital, Zhejiang University School of Medicine, Hangzhou, Zhejiang, China;
[26]Department of General Surgery, PLA General Hospital, Beijing, China;
[27]Westlake Laboratory of Life Sciences and Biomedicine, Westlake University, Hangzhou, Zhejiang, China;

[#]Co-first authors;
[*]Correspondence: Stan.ZQ.Li@westlake.edu.cn (S.Z.L.); kairos712@singnet.com.sg (O.L.K.); gmsngi@nus.edu.sg (N.G.I.); guotiannan@westlake.edu.cn (T.G.).

Supplementary figures

Supplementary Figure S1

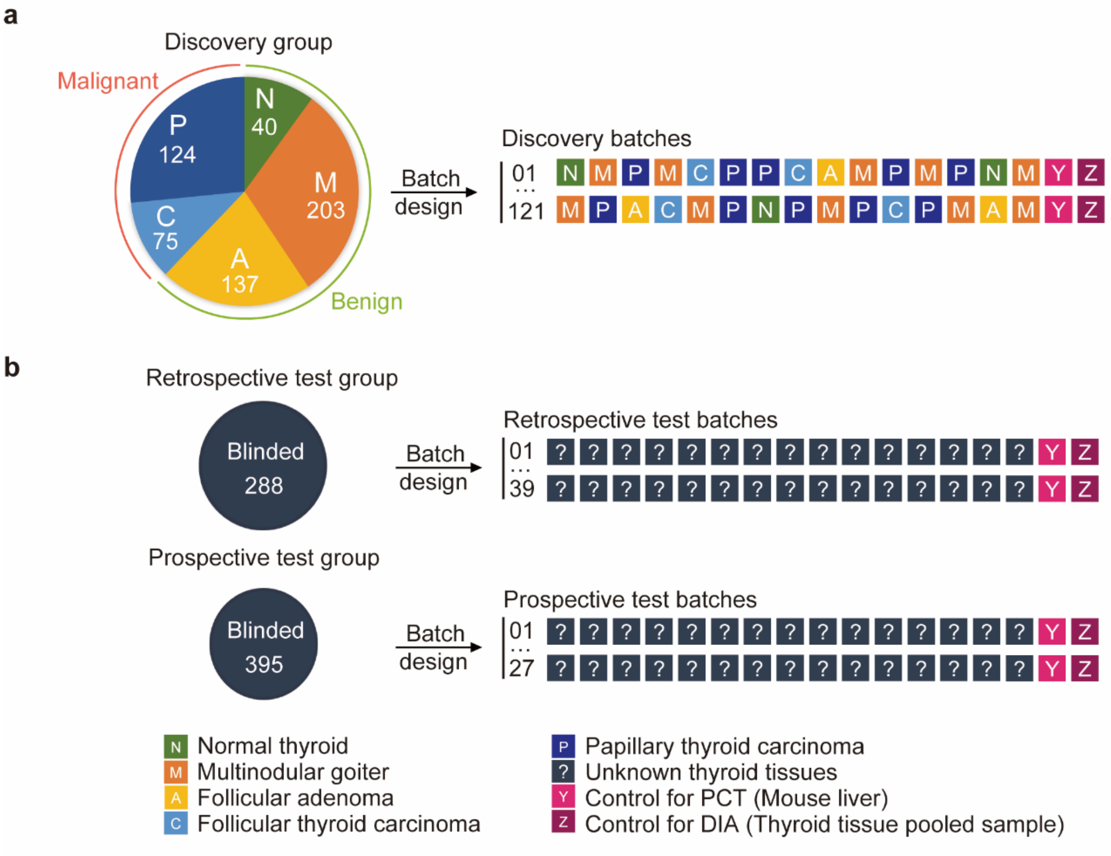

Supplementary Fig. S1. Batch design.

(**a**) The discovery group of 579 thyroid nodules (two nodules were excluded due to incorrect histological tissue type) from 578 patients consisted of 40 normal thyroid, 203 multinodular goiter, 137 follicular thyroid adenoma, 75 follicular thyroid carcinoma, and 124 papillary thyroid carcinoma samples with unblinded diagnoses. Three cores represented each nodule as biological replicates. Thyroid FFPE samples and technical replicates were randomly allocated into 121 discovery batches to minimize the batch effect for this large-scale sample preparation. (**b**) The independent test datasets comprised a retrospective test group and a prospective test group. The

retrospective test group comprised 288 thyroid nodules of blinded diagnoses from 271 patients. Each nodule was analyzed in technical duplicates, without biological replicates. A total of 288 FFPE cores and 288 corresponding technical duplicates were divided into 44 batches for analysis. The prospective test group contained 395 fine-needle aspiration biopsies of thyroid nodules which were divided into 27 batches. Each batch consisted of 15 thyroid samples, one mouse liver sample, and one pooled thyroid sample.

Supplementary Figure S2

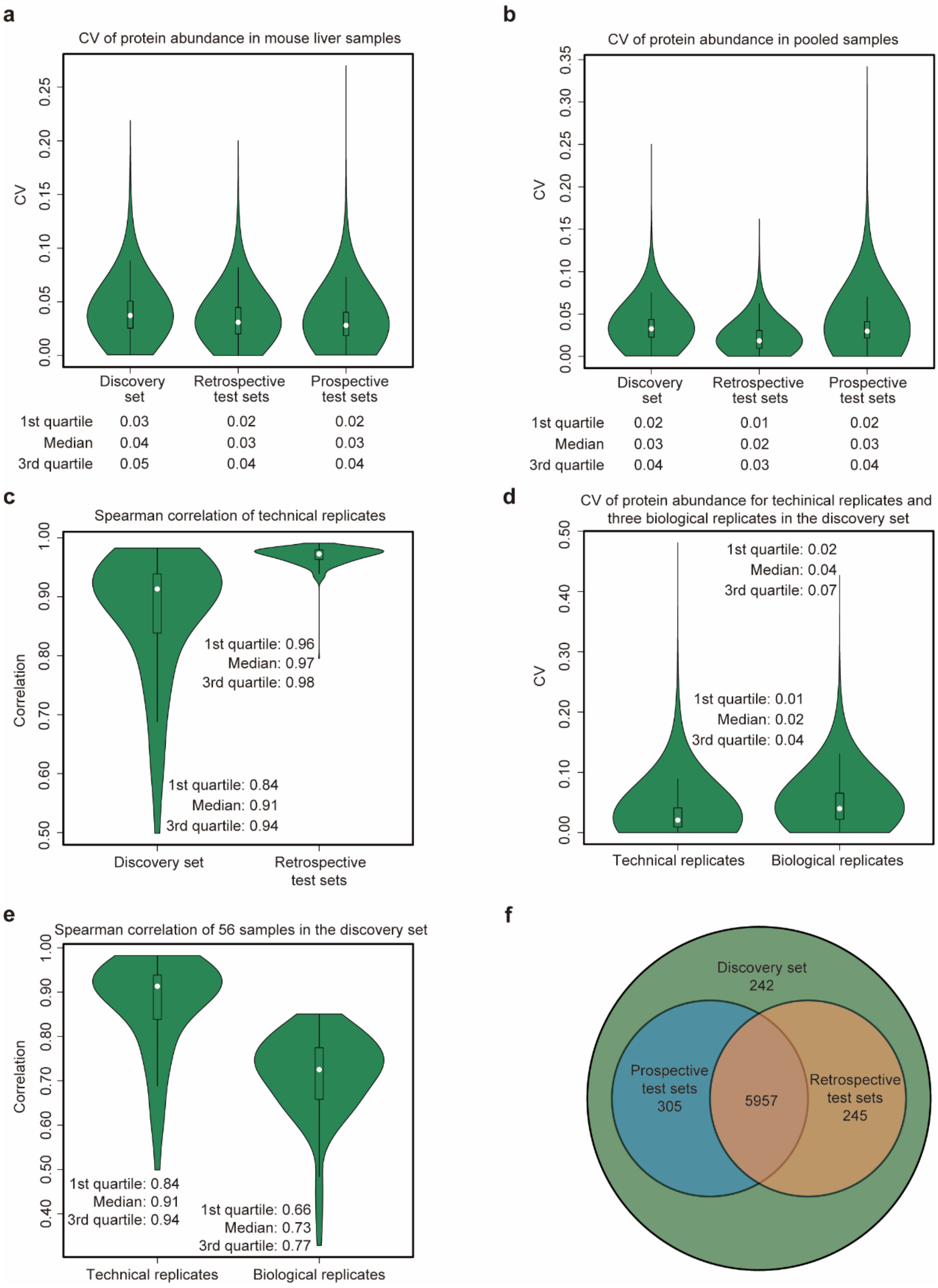

Supplementary Fig. S2. Data quality evaluation.

(**a**) Coefficient of variation (CV) of quantified protein abundance for 117, 36, and 27 pooled thyroid samples in the discovery set, retrospective, and prospective test sets, respectively. (**b**) CV of identified protein numbers for 112, 18, and 18 mouse liver samples in the discovery set, retrospective, and prospective test sets, respectively. (**c**) Spearman correlation of paired technical replicates from 56 randomly selected thyroid samples in the discovery set and 288 in the retrospective test sets. (**d**) CV for the number of proteins in technical and biological replicates of the discovery set. (**e**) Spearman correlation of paired technical replicates and biological replicates from 56 randomly selected thyroid samples in the discovery set. (**f**) Overlap of identified proteins in the three datasets. Altogether 5957 proteins were quantified.

Supplementary Figure S3

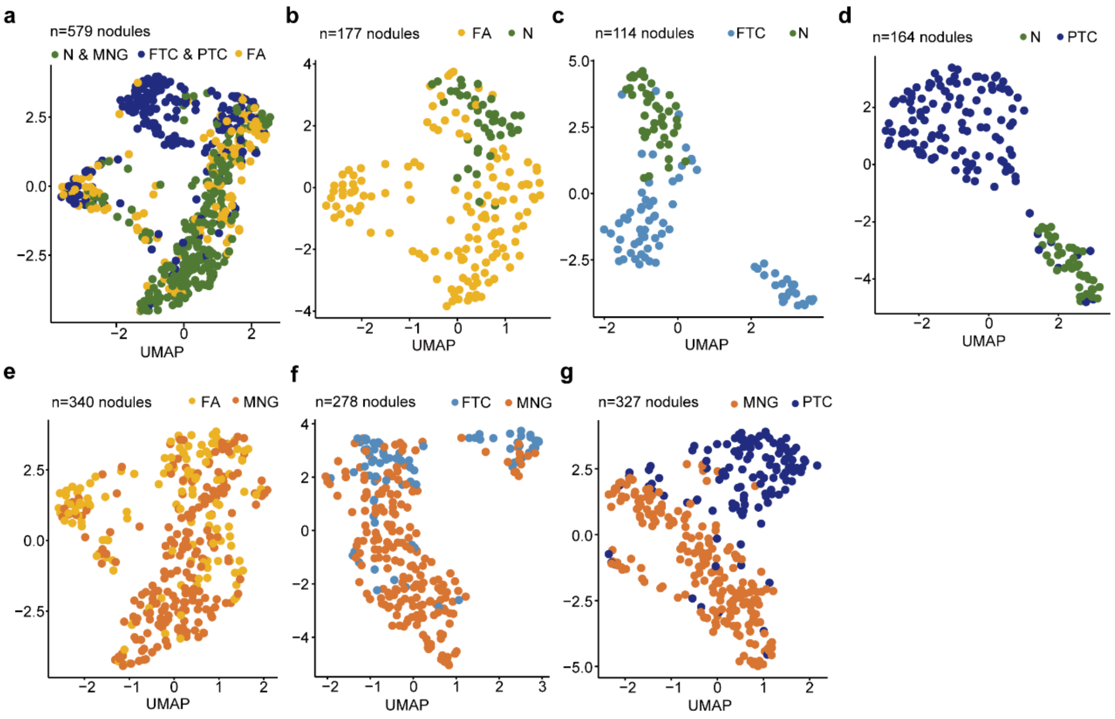

Supplementary Fig. S3. Uniform manifold approximation and projection (UMAP) analysis of five histotypes of thyroid tissues.

5312 proteins for which missing values were less than 90% were used in data analysis. (**a**) All tissue types, showing FA distributed across benign (N and MNG) and malignant (FTC and PTC) tissues, (**b**) FA *vs.* N; (**c**) FTC *vs.* N; (**d**) PTC *vs.* N; **(e)** FA *vs.* MNG; (**f**) FTC *vs.* MNG; and (**g**) PTC *vs.* MNG. Normal tissue was generally well separated from all other lesional tissues, while MNG showed some overlap with FA, FTC, and PTC.

Supplementary Figure S4

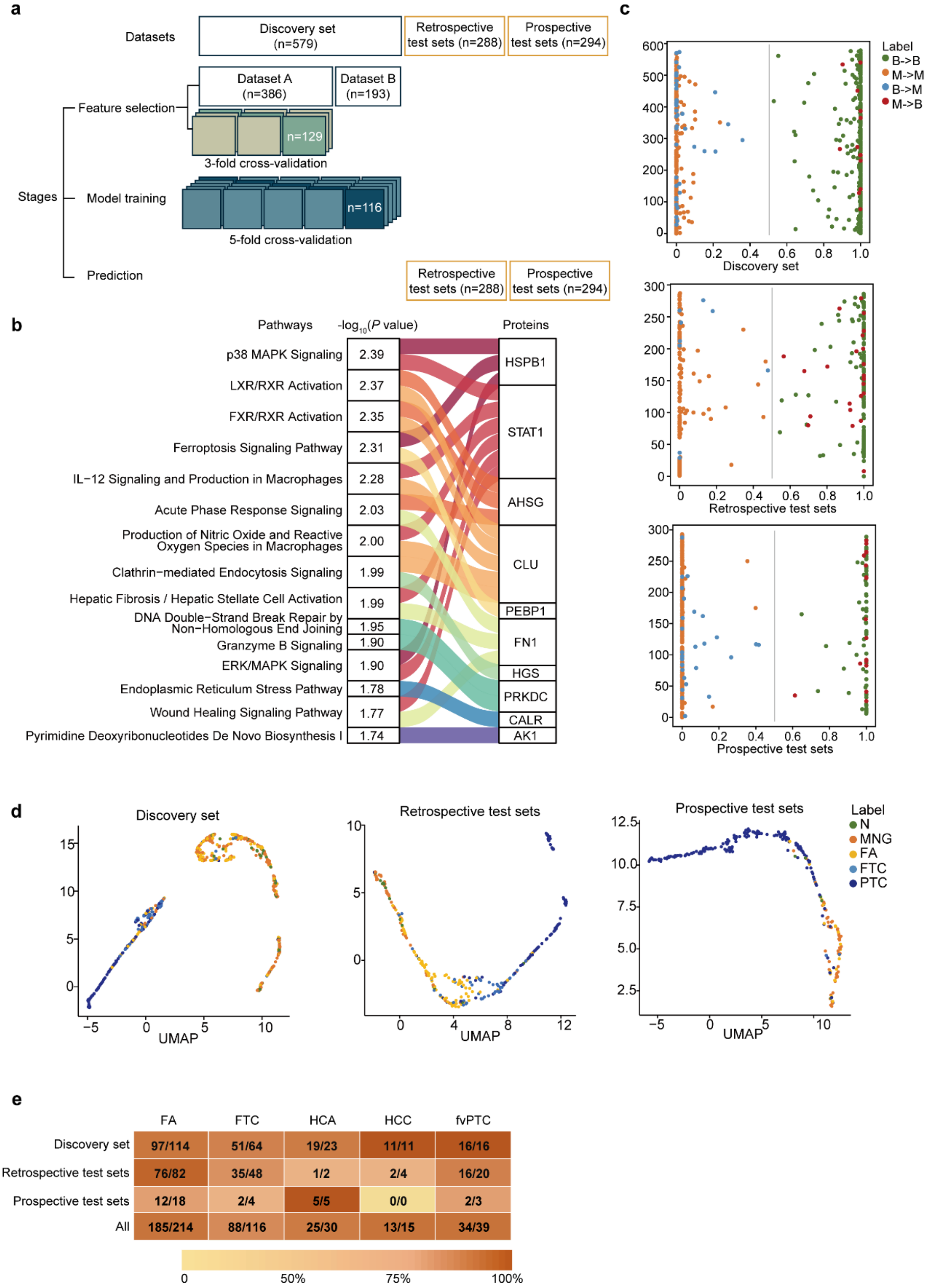

Supplementary Fig. S4. Data splitting, biological insights of selected features and cross-validation of the classifier on discovery set and performance on test sets. (**a**) Data splitting. (**b**) Pathway enrichment for the selected protein features. Sankey diagram showing the relationship between enriched pathways and corresponding proteins. *P* values were calculated by right-tailed Fisher's exact test through IPA software. (**c**) Scatter diagram showing the predicted malignancy scores for discovery (training and validation), and test sets (retrospective and prospective test sets), X-axis indicates the score for each sample. Score of 0.5 is the threshold for benign and malignant classification. Nodules with score more than 0.5 would be regarded as benign tissue Y-axis represents the number of thyroid tissues in different sets. (**d**) UMAP plots showing specific tissue types (benign and malignant) based on the 19 protein features in the training set, validation set, and retrospective and prospective test sets, labeled by each of the five histotypes. (**e**) Overall performance metrics of prediction of the neural network model for five follicular-pattern thyroid tumors per set. Graduated colors in the shaded bar indicate accuracy levels. Numbers in the boxes indicate the number of correctly identified samples/total sample number.

Supplementary Figure S5

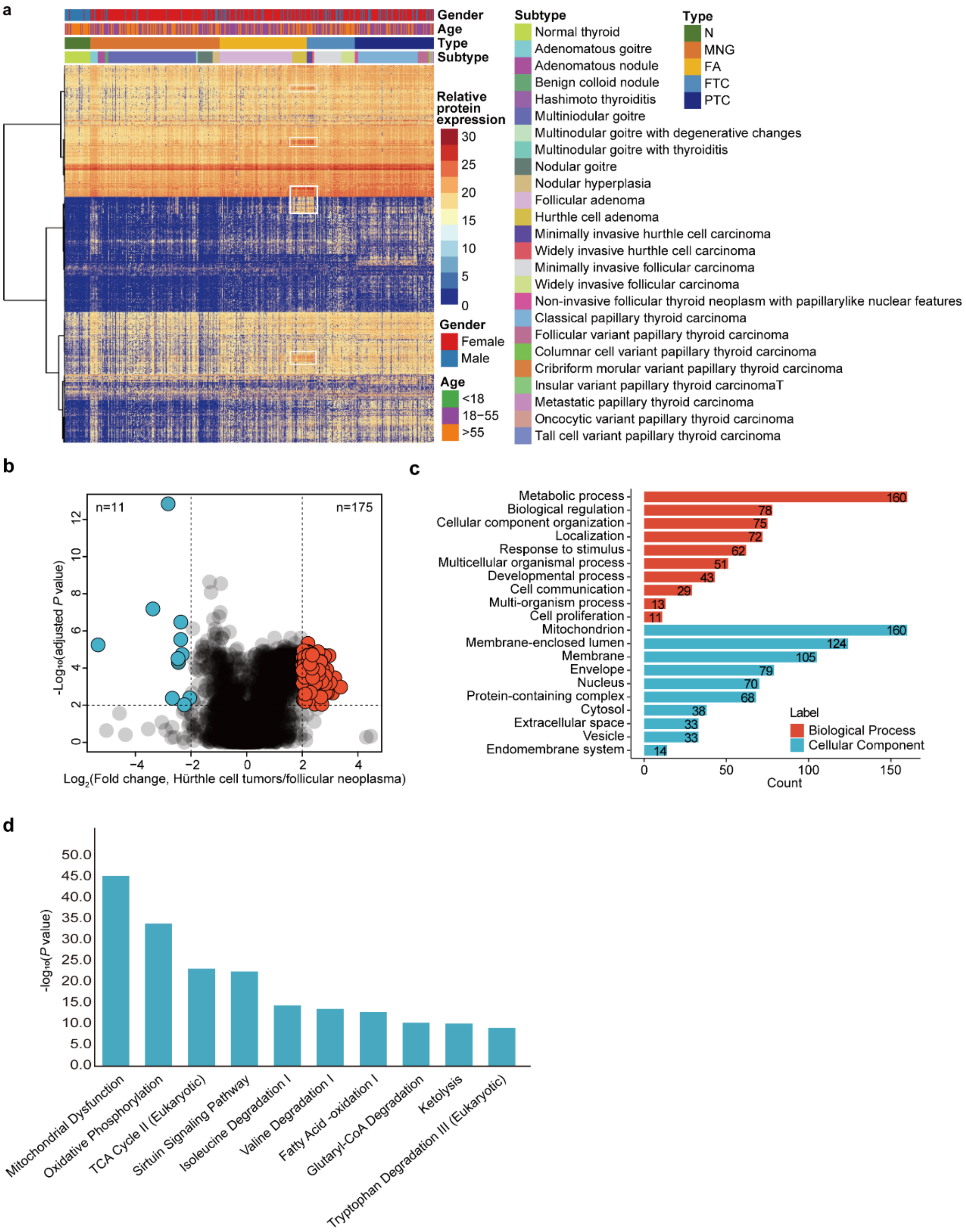

Supplementary Fig. S5. Biological insights into Hürthle cell tumors.

(**a**) Heatmap showing proteotype expression of thyroid tissue samples highlighting differentially expressed proteins in Hürthle cell neoplasms marked in white frames. Proteins were clustered by method of Ward.D1 in R package of pheatmap. An experienced histopathological reviewer assigned histological subtypes labeled in the heatmap. (**b**) Volcano plots showing differentially expressed proteins between Hürthle cell neoplasms vs. follicular neoplasms. Protein intensities used were the average intensity of three biological replicates. Proteins highlighted in red (up-regulated) or blue (down-regulated) were significantly different with a four-fold-change cutoff and adjusted *P* value threshold less than 0.01. (**c**) Graph showing gene ontology (GO) analysis of the 186 dysregulated proteins in Hürthle cell neoplasms. Mitochondrial proteins (160/186) were the most dominant group and most proteins mapped to the metabolic process. (**d**) Graph showing enriched top-ten pathways based on 186 dysregulated proteins of Hürthle cell tumors by IPA analysis. Y-axis shows $-\log_{10}$ (*P* value) based on right-tailed Fisher's exact test based on the IPA database.

Supplementary Figure S6

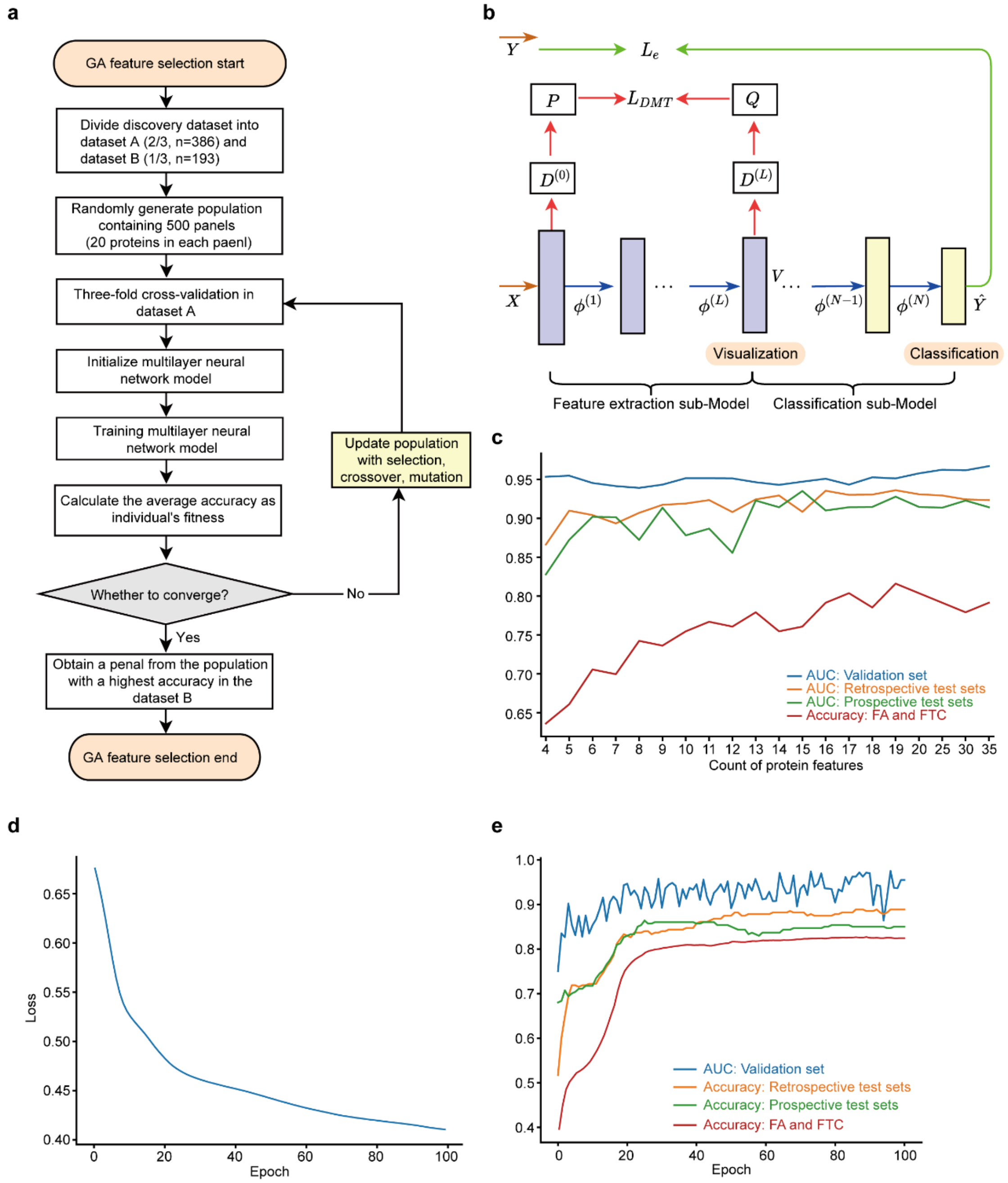

Supplementary Fig. S6. Structures of models and stability of the training algorithm.

(**a**) Flow diagram of genetic algorithm for protein features selection. (**b**) Structural diagram of neural network. (**c**) line chart showing the model performance on different counts of protein features. (**d**) Plots showing the model performance on loss, (**e**) AUC and accuracy for each epoch.

Supplementary Notes

Supplementary Note 1. Determinate the missing value threshold

In process of feature selection, we explored different screening conditions, using 25%,30%,35%, and 40% as thresholds for the missing value. The data (**Supplementary Table S5**) showed that at more relaxed screening conditions the higher number of candidate features were nominated and that the results became more unstable in the independent validation set. The features with higher missing value rates, although potentially providing better classification were not stably detected and may negatively affect the quality of the model if such features are selected. Finally, in our classifier, we clearly defined the criteria for feature selection and used 767 proteins that were missing in less than 35% of samples.

Supplementary Note 2. Model performance on different counts of features

To determine the count of protein features in the panel, we compare the model performances on the different number of features from four to 35 proteins. The more features the better accuracy and AUC achieved and reached a plateau when using 13 proteins (**Supplementary Table S6**). But for the most similar two histopathology types, FA and FTC, 19 proteins achieved the highest accuracy. Therefore, we used 19 proteins as a panel in the present study.

Supplementary Note 3. Evaluate the stability of selected features

Furthermore, we evaluated the stability of selected features by using 15 different

seeds (integers from 0 to 14) for 15 replicate GA experiments. In the GA stage, a good feature combination is defended as better than or equal to our proposed combination, $F^C \geq 0.917, \text{accuracy (validation set)} \geq 0.862$). While we reported a feature combination with high AUC, more than 94% in retrospective test datasets and 93% in prospective test datasets, the other selected feature combinations had high individual protein overlap with the reported (**Supplementary Table S7**). Therefore, the results indicate that the selected markers were stable predictors.

Supplementary Note 4. Three feature selection methods comparison

Additionally, we compared two frequently used feature selection methods in machine learning, LASSO, and Random Forest, with GA in two ways. The accuracy values from both alternative feature selection methods were lower than the GA, indicating that the GA yields better sets of features (**Supplementary Table S8**).

Supplementary Note 5. Model stability analysis

To visualize the stability of the training algorithm, we plotted the model performance on loss, AUC, and accuracy for each epoch. The curve of the loss function (**Supplementary Fig. S6d**) was stable and convergent, and the curve of accuracy (**Supplementary Fig. S6e**) did not fluctuate, further consolidating the stability of the model.

Supplementary Note 6. Different models comparison

Furthermore, we compared six alternative machine learning models with our established classification model using the 19 selected proteins (**Fig. 3e**). The protein panel was not optimized for each of the test classifiers, including our designed classifier. Logistic regression did not use L1 regularization (LASSO constraint) and was processed using scikit-learn standard logistic regression. Our model performed the best (AUC=0.93) followed by Random Forest (AUC=0.91), Logistic Regression (AUC=0.91), LASSO (AUC=0.91), MLP Classifier (AUC=0.91), Support Vector Machine (SVM, AUC=0.91) and Decision Tree (AUC=0.59).

Supplementary Note 7. Processing the unbalanced data

We designed the cross-entropy loss function by giving different weights to the two categories to deal with the imbalanced data. β_1 and $\beta_2 = 2 - \beta_1$ were the hyperparameters of the cross-entropy loss function. The table shows the model performance with different alpha values and alpha=1.6 achieved the highest AUC on the validation set, which was selected in the model.

Supplementary Tables

Supplementary Table S1. Detailed patient information of 1161 nodules

Supplementary Table S2. Protein matrices of discovery set, retrospective test sets, and prospective test sets

Supplementary Table S3. Detailed prediction results

Supplementary Table S4. Model performance of the 19-protein classifier on different sets

	**Discovery study**	**Retrospective study**	**Prospective study**	**Bethesda III and IV in the prospective study**	**Bethesda III in the prospective study**	**Bethesda IV in the prospective study**
Sample type	FFPE	FFPE	FNA biopsy	FNA biopsy	FNA biopsy	FNA biopsy
Total nodules (n)	579	288	294	74	52	22
Malignant nodules (n)	194	144	200	40	30	17
Benign nodules (n)	385	144	94	19	22	5
Prevalence (%)[a]	33.51 (29.78 - 37.45)	50.00 (44.26 - 55.74)	68.03 (62.49 - 73.10)	63.51 (52.13 - 73.56)	57.69 (44.19 - 70.13)	77.27 (56.56 - 89.88)
Predict M/M[b]	181	121	183	40	24	14
Predict B/B[c]	347	135	67	19	16	5
Sensitivity (%)	93.30 (88.87 - 96.04)	84.03 (77.17 - 89.11)	91.50 (86.81 - 94.63)	85.11 (72.31 - 92.59)	80.00 (62.69 - 90.50)	94.12 (73.02 - 98.95)
Specificity (%)	90.13 (86.74 - 92.72)	93.75 (88.55 - 96.68)	71.28 (61.44 - 79.45)	70.37 (51.52 - 84.15)	63.64 (42.95 - 80.27)	100.00 (56.55 - 100.00)
PPV (%)	96.39 (94.52 - 97.62)	85.44 (80.87 - 89.03)	79.76 (74.98 - 84.11)	73.08 (61.91 - 81.77)	70.00 (55.73 - 80.09)	83.33 (61.48 - 92.69)
NPV (%)	82.65 (79.44 - 85.59)	93.08 (89.52 - 95.46)	87.14 (82.76 - 90.44)	83.33 (73.76 - 90.47)	75.00 (61.79 - 84.77)	100.00 (85.13 - 100.00)
Accuracy (%)	91.19 (88.60 - 93.24)	88.89 (84.74 - 92.02)	85.03 (80.51 - 88.66)	79.73 (69.21 - 87.31)	73.08 (59.75 - 83.23)	95.45 (78.20 - 99.19)

Each value was calculated to 95% Wilson confidence intervals.

[a]The ratio of carcinoma in total nodules. The ratio of carcinoma in total nodules.

[b]The number of malignant nodules was correctly predicted as malignant.

[c]The number of benign nodules was correctly predicted as benign.

Supplementary Table S5. Model performance on different missing value rate features

Missing rate	Features	Overlap rate	AUC (Retrospective sets)	AUC (Prospective sets)
30%	P02765, P04083, O00339, P04899, O75347, P04216, P02751, P26038, P00568, P78527, P04792, P35579, P42224, P27797, Q9HAT2, P46940, O14964, Q8IXM2, P17931	0.74	0.92	0.92
	P02765, P04083, O00339, P04899, O75347, P04216, P02751, P26038, P00568, P78527, P04792, P35579, P42224, P27797, Q9HAT2, P46940, O14964, Q8IXM2, Q01130	0.68	0.90	0.90
	P02765, P04083, O00339, P04899, O75347, P04216, P02751, P26038, P00568, Q13263, P15090, Q8WX93, P42224, Q9Y696, Q9HAT2, P30086, O14964, P10909, P17931	0.68	0.93	0.91

	P02765, P04083, O00339, P04899, O75347, P23297, P02751, P26038, P00568, P78527, P04792, P35579, Q7Z4V5, P27797, O00170, Q12797, O14964, P09467, P17931	0.58	0.86	0.89
	P02765, P04083, O00339, P04899, O75347, P23297, P02751, P26038, P00568, P78527, P04792, P35579, Q7Z4V5, P27797, Q9BUT1, Q12797, O14964, P09467, P17931	0.58	0.89	0.92
	P02765, P04083, O00339, P04899, O75347, P23297, P02751, P26038, P00568, P78527, P04792, P35579, Q7Z4V5, P27797, Q9Y5X3, Q12797, O14964, P09467, P17931	0.58	0.88	0.91
	P02765, P06703, O00339, P04899, P37802, P04216, P09496, P26038, P00568, P15090, Q07654, Q6IQ23, P25788, P50402, Q9HAT2, P30086, O43143, P10909, P17931	0.42	0.89	0.87

	P02765, P06703, O00339, P04899, P37802, P04216, P09496, P26038, P00568, P15090, Q07654, Q6IQ23, P25788, P50402, Q9HAT2, P30086, O43143, P10909, P17931	0.42	0.93	0.93
	P02765, P07202, O00339, P04899, O75347, P04216, P02751, P26038, P00568, P78527, P04792, P35579, P42224, P27797, Q9HAT2, P30086, P20340, P10909, P17931	0.74	0.91	0.93
	P50454, P04083, O00339, P04899, O75347, P04216, P02751, P06703, P00568, P78527, P04792, P35579, P42224, P27797, Q9HAT2, P30086, O14964, P10909, P17931	0.79	0.93	0.92
**35% (Selected)**	**P02765, P04083, O00339, P58546, O75347, P04216, P02751, P83731, P00568, P78527, P04792, P57737, P42224, P27797, Q9HAT2, P30086, O14964, P10909, P17931**	**--**	**0.94**	**0.93**
40%	P02765, P04083, O00339, P04899, O75347, P04216, P02751, P82979,	0.84	0.91	0.89

	P00568, P78527, P04792, P35579, P42224, P27797, Q9HAT2, P30086, O14964, P10909, P17931			

Supplementary Table S6. Model performance on different counts of features

Feature count	AUC (Validation sets)	AUC of (Retrospective sets)	AUC (Prospective sets)	Accuracy (FA and FTC)
4	0.954	0.866	0.828	0.636
5	0.955	0.910	0.872	0.661
6	0.946	0.904	0.902	0.706
7	0.942	0.894	0.902	0.699
8	0.939	0.907	0.872	0.742
9	0.943	0.917	0.914	0.736
10	0.952	0.919	0.878	0.755
11	0.952	0.924	0.887	0.767
12	0.951	0.908	0.856	0.761

13	0.947	0.924	0.923	0.779
14	0.943	0.929	0.914	0.755
15	0.947	0.908	0.935	0.761
16	0.951	0.936	0.910	0.791
17	0.943	0.930	0.914	0.804
18	0.953	0.931	0.915	0.785
**19 (Selected)**	**0.951**	**0.936**	**0.928**	**0.816**
20	0.958	0.931	0.915	0.804
25	0.963	0.929	0.914	0.791
30	0.962	0.924	0.923	0.779
35	0.967	0.924	0.914	0.791

Supplementary Table S7. Model performance on different seeds for GA

**Seed**	**Features**	**AUC (Retrospective sets)**	**AUC (Prospective sets)**
**Our model**	**P02765, P04083, O00339, P58546, O75347, P04216, P02751, P83731, P00568, P78527, P04792, P57737, P42224, P27797, Q9HAT2, P30086, O14964, P10909, P17931**	**0.94**	**0.93**
1	P02765, P04083, O00339, P04899, O75347, P04216, P02751, P26038, P00568, P05109, Q16643, P35579, P42224, P09211, Q9HAT2, P30086, O14964, P10909, P17931	0.90	0.92
4	P02765, P04083, O00339, P58546, O75347, P04216, P02751, P83731, P00568, P78527, P04792, P57737, P42224, P27797, Q9HAT2, P30086, O14964, P10909, P17931	0.94	0.93

7	O43290, P02766, O00339, Q14498, Q04917, P04216, P02751, P26038, P61978, P35579, P43405, P08962, P68366, P06753, Q96HE7, P08727, O75521, Q9Y3F4, P17931	0.86	0.85
	P02765, P04083, O00339, P04899, O75347, P04216, P02751, P08727, P00568, P78527, P04792, P35579, P42224, P27797, Q9HAT2, P30086, O14964, P10909, P17931	0.92	0.91
8	P08238, P02766, O00339, P02765, Q04917, P04216, P02751, P09543, P61978, P35579, P43405, P68871, P68366, P06753, Q96HE7, P08727, O75521, Q9Y3F4, P17931	0.90	0.91
	P08238, P02766, O00339, P02765, Q04917, P04216, P02751, P17612, P61978, P35579, P43405, P68871, P68366, P06753, Q96HE7, P08727, O75521, Q9Y3F4, P17931	0.87	0.88

	P08238, P02766, O75369, P02765, Q15149, P02511, P02751, P17612, P61978, P35579, P43405, P68871, P51580, P06753, Q96HE7, P39059, O75521, Q13263, P17931	0.88	0.82
11	P02765, P04083, O00339, P04899, O75347, P04216, P02751, P82979, P00568, P78527, P04792, P35579, P42224, P27797, Q9HAT2, P30086, O14964, P10909, P17931	0.90	0.89
12	P02765, Q8WX93, P27824, P04899, Q9HCD5, P04216, Q53EL6, P26038, Q9NP61, P04080, P30837, P35579, Q8NHG8, P07202, Q9HAT2, P30086, O60934, P10909, P17931	0.90	0.82

Supplementary Table S8. Model performance on different feature selection methods and models

Features	Feature count	Accuracy (Retrospective sets)	Accuracy (Prospective sets)
**Our model**	**19**	**0.89**	**0.85**
**LASSO selected features with lasso model**			
P01903, P07202, P09758, P16671, P22748, P27487, P35625, P36021, P52926, P58107, Q07654, Q07817, Q13509, Q15742, Q16656, Q6IQ23, Q8IV56, Q8WUF5, Q8WXX5, Q9BV79, Q9BWM7, Q9NX55, Q9P2K5	23	0.79	0.76
P01903, P07202, P09758, P16671, P22748, P27487, P35625, P52926, P58107, Q07654, Q07817, Q13509, Q15742, Q16656, Q6IQ23, Q8IV56, Q8WUF5, Q8WXX5, Q9BV79, Q9BWM7, Q9NX55, Q9P2K5	22	0.80	0.77
P01903, P07202, P09758, P16671, P22748, P27487, P35625, P52926,	21	0.79	0.77

P58107, Q07654, Q07817, Q13509, Q15742, Q16656, Q6IQ23, Q8IV56, Q8WUF5, Q8WXX5, Q9BV79, Q9BWM7, Q9P2K5			
P01903, P07202, P09758, P16671, P22748, P27487, P35625, P52926, P58107, Q07654, Q13509, Q15742, Q8IV56, Q8WUF5, Q8WXX5, Q9BV79, Q9BWM7, Q9P2K5	18	0.80	0.78
P01903, P07202, P09758, P16671, P22748, P27487, P35625, P52926, P58107, Q07654, Q13509, Q15742, Q8WUF5, Q8WXX5, Q9BV79, Q9P2K5	16	0.81	0.78
**LASSO selected features with our model**			
P01903, P07202, P09758, P16671, P22748, P27487, P35625, P36021, P52926, P58107, Q07654, Q07817, Q13509, Q15742, Q16656, Q6IQ23, Q8IV56, Q8WUF5, Q8WXX5, Q9BV79, Q9BWM7, Q9NX55, Q9P2K5	23	0.83	0.81
P01903, P07202, P09758, P16671, P22748, P27487, P35625, P52926, P58107, Q07654, Q07817, Q13509, Q15742, Q16656, Q6IQ23, Q8IV56,	22	0.84	0.81

Q8WUF5, Q8WXX5, Q9BV79, Q9BWM7, Q9NX55, Q9P2K5			
P01903, P07202, P09758, P16671, P22748, P27487, P35625, P52926, P58107, Q07654, Q07817, Q13509, Q15742, Q16656, Q6IQ23, Q8IV56, Q8WUF5, Q8WXX5, Q9BV79, Q9BWM7, Q9P2K5	21	0.83	0.80
P01903, P07202, P09758, P16671, P22748, P27487, P35625, P52926, P58107, Q07654, Q13509, Q15742, Q8IV56, Q8WUF5, Q8WXX5, Q9BV79, Q9BWM7, Q9P2K5	18	0.84	0.81
P01903, P07202, P09758, P16671, P22748, P27487, P35625, P52926, P58107, Q07654, Q13509, Q15742, Q8WUF5, Q8WXX5, Q9BV79, Q9P2K5	16	0.83	0.80
**Random Forest selected features with our model**			
O00154, O15020, O95171, P09668, P11137, P16989, P42167, P54727, P60660, Q00688, Q13185, Q14847, Q15149, Q15742, Q9BW04, Q9UBG0, Q9UJU6	17	0.73	0.76

O00154, O15020, O95171, O95436, P09668, P11137, P12268, P16989, P42167, P50479, P54727, P60660, Q00688, Q13185, Q14847, Q15149, Q15742, Q9BW04, Q9UBG0, Q9UJU6	20	0.75	0.75
O00154, O15020, O95171, O95436, P04083, P09668, P11137, P12268, P16989, P42167, P50479, P54727, P60660, P63313, Q00688, Q13185, Q14847, Q15149, Q15742, Q99961, Q9BW04, Q9BZG1, Q9UBG0, Q9UJU6	24	0.74	0.73
**Random Forest selected features with Random Forest model**			
O00154, O15020, O95171, P09668, P11137, P16989, P42167, P54727, P60660, Q00688, Q13185, Q14847, Q15149, Q15742, Q9BW04, Q9UBG0, Q9UJU6	17	0.77	0.76
O00154, O15020, O95171, O95436, P09668, P11137, P12268, P16989, P42167, P50479, P54727, P60660, Q00688, Q13185, Q14847, Q15149, Q15742, Q9BW04, Q9UBG0, Q9UJU6	20	0.73	0.71

O00154, O15020, O95171, O95436, P04083, P09668, P11137, P12268, P16989, P42167, P50479, P54727, P60660, P63313, Q00688, Q13185, Q14847, Q15149, Q15742, Q99961, Q9BW04, Q9BZG1, Q9UBG0, Q9UJU6	24	0.75	0.73

Supplementary Table S9. Model performance with different alpha values

**alpha**	**AUC (Validation sets)**	**AUC (Retrospective sets)**	**AUC (Prospective sets)**
1	0.9473	0.91	0.87
1.2	0.9512	0.91	0.91
1.4	0.9496	0.94	0.94
**1.6 (Selected)**	**0.9514**	**0.94**	**0.93**
